# Chronic stress in relation to clinical burnout: an integrative scoping review of definitions and measurement approaches

**DOI:** 10.3389/fpsyg.2025.1712340

**Published:** 2025-12-12

**Authors:** Rebecca Vandenabeele, Margot C. W. Joosen, Arno van Dam

**Affiliations:** 1 Tilburg School of Social and Behavioral Sciences, Tranzo Scientific Center for Care and Wellbeing, Tilburg University, Tilburg, Netherlands; 2 CSR Centre - Expertise centre for Stress and Resilience, Meerkerk, Netherlands; 3 GGZ Westelijk Noord Brabant, Halsteren, Netherlands

**Keywords:** clinical burnout, chronic stress, biomarkers, psychosocial, measurement, biopsychosocial model, system dynamics theory

## Abstract

**Background:**

Since its introduction, the definition and diagnosis of burnout remain controversial. Although the literature distinguishes between severe clinical burnout, characterized by debilitating symptoms and prolonged recovery, and milder burnout complaints in generally healthy employees, unclear diagnostic criteria hinder reliable differentiation. As clinical burnout is often framed as an outcome of chronic stress, clarifying how chronic stress is defined and measured is crucial to improve the understanding, diagnosis, and distinction of clinical burnout.

**Objective:**

This scoping review synthesizes results from systematic reviews using an integrative approach. It examines how chronic stress is defined and measured, from psychosocial, biological, and biopsychosocial perspectives. Additionally, it explores the discriminative value of these measurement approaches in identifying individuals at risk of chronic stress-related disorders and in diagnosing clinical burnout.

**Methods:**

A systematic search was performed on April 9th, 2024, and updated on July 8th, 2025 (PsychInfo, Embase, Medline and PubMed), following the PRISMA-ScR guidelines. The search identified 2,645 abstracts, 219 were included for full text screening, and 45 reviews were analyzed, covering over 2000 studies.

**Results:**

Across reviews, definitions of chronic stress varied, referring to either internal and external stressors or to stress responses. Measurements ranged from biomarkers and psychosocial scales to downstream health outcomes, such as burnout. In evaluating their discriminative value, chronic stress was consistently associated with HPA-axis dysregulation, immune impairment, autonomic imbalance, and elevated allostatic load, validating the biopsychosocial model of chronic stress. Sleep and cognitive deficits emerged as both causes, consequences, and maintaining factors of chronic stress. Among psychosocial instruments, the Burnout Assessment Tool (BAT) demonstrated the strongest psychometric properties, showing superior ability to distinguish individuals at risk of burnout from healthy controls. However, no single biomarker or questionnaire demonstrated sufficient accuracy to diagnose clinical burnout.

**Conclusion:**

No agreed-upon definition of chronic stress was found, nor is there consensus on how it should be measured. This lack of conceptual clarity hampers reliable diagnosis of clinical burnout. Progress in diagnosing and treating clinical burnout requires moving beyond single-cause diagnostics toward integrative biopsychosocial frameworks, such as system dynamics theory, that capture the complex, evolving nature of chronic stress and provide a foundation for more accurate diagnosis and treatment.

## 1 Introduction

Burnout is an increasingly prevalent syndrome associated with significant individual suffering, reduced societal productivity, and substantial economic costs due to decreased work performance and elevated healthcare utilization (Han et al., 2019; Neill et al., 2022; Martinez et al., 2025).

Burnout has been described in numerous ways since its initial introduction in the 1970s, resulting in considerable variability in its conceptualization, diagnosis, and treatment (Freudenberger, 1974; Ahola et al., 2017; Rotenstein et al., 2018). A systematic review by Guseva Canu et al. (2021) identified as many as 88 unique definitions of burnout across the scientific literature, reflecting the lack of consensus. Currently, burnout is classified by the World Health Organization (2019) and World Health Organization (2022) in the ICD-11 as an occupational phenomenon, defined as the result of “chronic workplace stress that has not been successfully managed.,” and not as a medical condition. Even so, no universally accepted terminology or diagnostic criteria for burnout exist, contributing to the ongoing debate regarding its clinical status (Weber and Jaekel-Reinhard, 2000; Bianchi et al., 2014; Nadon et al., 2022). This lack of consensus has recently intensified discussions around the conceptualization and classification of burnout. Bianchi and colleagues (Bianchi and Schonfeld, 2024, 2025) argue that burnout substantially overlaps with depression and may represent a specific subtype or manifestation of depressive disorders. In contrast, De Beer and Schaufeli (2025), De Witte and Schaufeli (2025), Demerouti and Bakker (2025), and Leiter and Day (2025) contend that burnout is a distinct construct, arising primarily from prolonged occupational stress and should be differentiated from clinical depression. This debate extends beyond definitional boundaries, touching on the question of whether burnout is exclusively job-related, how its increasing prevalence should be interpreted, and whether it merits recognition as a formal diagnostic category.

Even though burnout is commonly associated with prolonged or excessive exposure to stress, often in the workplace, the severity of symptoms can vary widely. Most burnout research has focused on relatively healthy employees who report burnout complaints through self-rated burnout scales. These individuals often show a reduction in burnout scores once stressors are alleviated or when interventions based on cognitive-behavioral therapy (CBT), mindfulness-based interventions (MBI), and complementary self-care strategies are implemented (Tamminga et al., 2023; Sharin et al., 2025). In contrast, a small but distinct subgroup experiences severe and enduring symptoms, with recovery periods exceeding 1 year (van der Klink et al., 2003; Van Dam et al., 2012; Eskildsen et al., 2016; Van Dam, 2016) with some even experiencing reduced stress tolerance and cognitive impairments up to 2–7 years after diagnosis (Van Dam et al., 2012; Osterberg et al., 2014; Oosterholt et al., 2016; Bjork et al., 2018; Glise et al., 2020). As a result, these affected individuals continue to seek support from healthcare professionals, with stress-related complaints frequently contributing to extended sickness absence and significant occupational health burden. In an attempt to describe the complexity of burnout in relation this severe form of exhaustion and prolonged recovery, various terms have emerged in the literature, such as clinical burnout (Van Dam, 2021), severe burnout or burnout disorder (Schaufeli et al., 2023a; Schaufeli et al., 2023b), and exhaustion due to non-traumatic stress (van de Leur et al., 2020). In response to rising stress-related sick leave, Sweden introduced Exhaustion Disorder (ED) as a medical diagnosis in 2003 (The National Board of Health and Welfare, 2003), later included in the Swedish ICD-10 (code F43.8A) to describe severe, prolonged stress-related exhaustion that largely overlaps with clinical burnout (Van Dam, 2021). For improved readability,in this review, the term clinical burnout will be used to refer to the severe form of burnout involving debilitating symptoms and extended recovery time, arising from chronic stress that may stem from both work-related and private stressors.

The lack of a universally agreed-upon definition and standardized diagnostic criteria for clinical burnout as a disorder related to chronic stress, presents a significant challenge for both research and clinical practice, underscoring the need for a standardized framework to improve diagnosis, treatment, and prevention strategies.

Despite the lack of consensus on how clinical burnout is defined, there is general agreement that chronic stress, whether work-related or stemming from personal life stressors, plays a key role in triggering the debilitating symptoms associated with clinical burnout (Khammissa et al., 2022; Vinkers, 2022; Bianchi and Schonfeld, 2024; De Beer and Schaufeli, 2025). Understanding how chronic stress is defined and measured, is therefore essential to advancing both clinical practice and research in this field. This need is underscored by the broader body of research showing that chronic stress is not only implicated in burnout but also constitutes a major global contributor to mental and physical health problems. According to the World Health Organization (2022), over 970 million people suffer from mental or neurological disorders, many of which are stress related. While acute stress responses serve adaptive functions, prolonged or chronic stress has been consistently linked to adverse health outcomes, including depression, anxiety, and burnout (Marin et al., 2011), as well as cardiovascular disease, metabolic disturbances, and autoimmune dysfunction (McEwen, 1998; O'Connor et al., 2021). Despite the extensive research linking chronic stress to negative health outcomes, there is currently no universally accepted definition of what constitutes chronic stress. In the context of clinical burnout, the absence of a shared understanding of chronic stress presents a critical gap. This lack of definitional clarity hampers progress in identifying at-risk populations, comparing outcomes across studies, and designing effective interventions. Given the overlap between chronic stress and burnout, developing a coherent conceptual and measurement framework for chronic stress may help inform future efforts to define and diagnose clinical burnout more precisely.

Stress research has a long and diverse history across biological, psychosocial, and biopsychosocial fields, each adopting distinct approaches. Recognizing this background is essential for understanding how stress has been defined and measured over time. Since the study of stress began, research has shown it to be a multifaceted concept, engaging emotional, cognitive, and biological systems in complex ways. Early 20th-century, stress research was approached mainly from a biological perspective, defining it as the body’s attempt to maintain internal stability (homeostasis) in response to nonspecific stressors (Cannon, 1932; Selye, 1946; Bernard, 1974). In this view, stress was considered a neutral, nonspecific reaction of the body to demands, studied mainly through endocrine and immune changes. It was highlighted how acute stress could be adaptive, but that chronic activation of the stress system led to exhaustion and physical strain, a process also described in Selye’s General Adaptation Syndrome, which outlines the stages of alarm, resistance, and eventual exhaustion in response to prolonged stress (Selye, 1946). From the 1960s onward, the focus shifted toward a psychosocial perspective, emphasizing the importance of subjective appraisal, personality traits, social support, and coping strategies in shaping an individual’s stress response. In other words, not the stressor itself but the subjective experience and appraisal of that stressor were considered to elicit a stress response, as demonstrated by the transactional model of stress (Lazarus, 1966). By the 1980s, researchers began integrating both physiological and psychosocial elements into a biopsychosocial perspective, introducing concepts such as allostatic load to explain how prolonged stress can lead to neuroendocrine alterations, particularly HPA-axis dysregulation, and immune system dysfunction that can ultimately result in serious health consequences (Sterling and Eyer, 1988; McEwen and Stellar, 1993). Today, a range of biomarkers, physiological indicators, and self-report measures are used to capture both the biological processes and the subjective experience of chronic stress.

Despite decades of research across biological, psychological, and social dimensions, the field still lacks a universally accepted definition of chronic stress and standardized parameters for its measurement. This ambiguity not only hinders the comparability of research findings but also limits the development of targeted interventions and public health strategies. Bridging this gap by establishing a comprehensive, integrative framework for defining and quantifying chronic stress is essential for translating scientific insights into effective clinical actions, particularly in the context of diagnosing stress-related conditions such as clinical burnout.

Since clinical burnout is fundamentally recognized as a chronic stress-related disorder, researchers have increasingly examined the link between clinical burnout and physiological dysregulation by focusing on biological stress-related systems, particularly the autonomic nervous system (ANS) and the hypothalamic–pituitary–adrenal (HPA) axis (Danhof-Pont et al., 2011; Jonsdottir and Sjors Dahlman, 2019). It is hypothesized that clinical burnout, as well as other forms of chronic stress, involves sustained activation of the ANS and dysregulation of the sympathetic-adrenal-medullary (SAM) and Hypothalamic–Pituitary–Adrenal (HPA) axes, leading to altered neuroendocrine, immune, and metabolic function, which may cause the detrimental health consequences related to chronic stress or clinical burnout (Chrousos and Gold, 1992; Lupien et al., 1998; Lupien et al., 2009; Ganzel et al., 2010; Ganzel and Morris, 2011; Golkar et al., 2014; McEwen, 2017; Epel et al., 2018; Godoy et al., 2018; Bayes et al., 2021; Roberts and Karatsoreos, 2021; Pihlaja et al., 2022; Alotiby, 2024). Over time, this prolonged stress activation can recalibrate the body’s set points to a higher baseline, a process recognized as allostatic load, in which HPA-axis dysregulation plays a key role (Sterling, 2004; McEwen, 2017). This not only affects the body’s biological processes but also influences mental well-being and social behavior (Sapolsky, 2007). According to the biopsychosocial perspective, such biological mechanisms could help explain the physical and mental symptoms experienced by those suffering from severe or clinical burnout (Ganzel and Morris, 2011; Bayes et al., 2021; Roberts and Karatsoreos, 2021).

The link between clinical burnout and chronic stress underscores the importance of clearly defining what chronic stress entails and establishing accurate measurement methods. Without a clear conceptualization of chronic stress, it is difficult to identify individuals at risk of developing clinical burnout, or design targeted interventionsGiven the broad and multidisciplinary nature of (chronic) stress research, encompassing biological, psychological, biopsychosocial and connected cognitive, emotional, and behavioral domains, a scoping review approach was deemed most appropriate. This design allowed us to systematically map and synthesize the diverse conceptualizations and measurement approaches within the heterogeneous field of chronic stress and burnout (Munn et al., 2018). This scoping review aims to systematically map and analyze the existing scientific literature on the conceptualizations, definitions, explanatory models, and measurement approaches of chronic stress, both within and beyond the context of (clinical) burnout. In addition, it examines the extent to which these measurement approaches demonstrate discriminative value and clinical applicability in identifying or diagnosing clinical burnout. Given the fragmentation of stress research across biological, psychological, and biopsychosocial perspectives, this review seeks to clarify how chronic stress is conceptualized and assessed across these domains. The insights gained may advance understanding of clinical burnout and inform efforts to enhance its definition and diagnosis. Research questions:

1. Conceptualization: What definitions and conceptualizations of chronic stress, whether or not in the context of burnout, are used in the scientific literature?
2. Explanatory Models: Which explanatory models, fitting with the biological, psychosocial and/or biopsychosocial perspective, are used to link chronic stress to adverse health outcomes?
3. Measurement approaches: What measurement parameters or tools, ranging from physiological biomarkers to psychosocial indicators, are reported to assess chronic stress?
4. Discriminative value: To what extent do current chronic stress measurement tools demonstrate discriminative value and clinical usability in diagnosing clinical burnout specifically?

## 2 Methods

### 2.1 Study design

**Figure 1:**
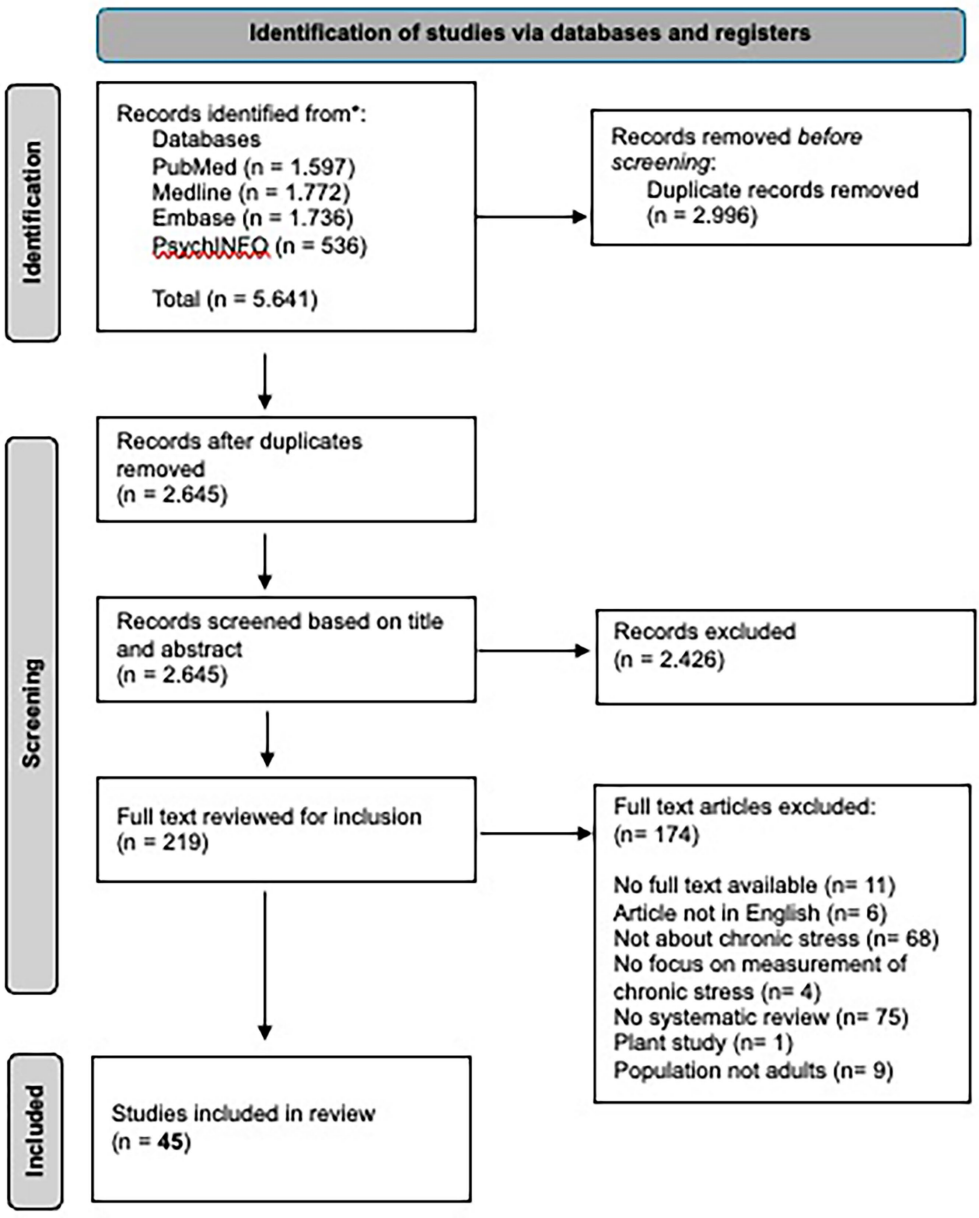
PRISMA flow chart outlining the systematic search strategy for the identification and selection of reviews (Page et al., 2021).

We conducted this scoping review following the guidelines for scoping reviews from the Joanna Briggs Institute (Peters et al., 2020) and adhered to the Preferred Reporting Items for Systematic Reviews and Meta-Analyses extension for Scoping Reviews (PRISMA-ScR) checklist (Tricco et al., 2018), including standards for reporting, inclusion, screening, data extraction, and synthesis. Although not a systematic review, a PRISMA flowchart was included to enhance transparency in the article selection process (Page et al., 2021) (see Figure 1). Although the review followed the PRISMA-ScR guidelines, no formal protocol was registered.

### 2.2 Eligibility criteria

Reviews were included if they met the following criteria: (1) focus on chronic stress or burnout as a conceptualization of chronic, persistent or prolonged stress; (2) rendition on how chronic stress is measured or assessed from a biological, psychosocial, or biopsychosocial perspective, including the use of (3) biomarkers, indicators, heart rate variability, or psychometric questionnaires or surveys; (4) being (systematic) reviews or meta-analyses. Only reviews that employed a clearly defined and systematic search methodology were included. That is, more than one database was searched, with a stated search strategy and inclusion- and exclusion-criteria were described. We considered only peer-reviewed full text articles published in English. We included published research from all years to maximize the scope of our review. Articles that did not meet these criteria, such as books, empirical studies, randomized control trial studies, dissertations, or reviews presenting only theoretical perspectives or author opinions, were excluded. Furthermore, we only selected articles involving adult human subjects of any health status.

### 2.3 Search strategy and information sources

We conducted the search on April 9, 2024, and updated it on July 8th, 2025, across four databases: PubMed, MEDLINE, Embase, and PsycINFO via EBSCOhost.

To address our research questions, we developed a comprehensive search strategy grounded in four key elements: (1) chronic stress and burnout; (2) perspectives (biological, psychosocial, and biopsychosocial); (3) measurements; and (4) type of research. An experienced information analyst assisted in formulating the search strings using relevant keywords and controlled vocabulary (Medical Subject Headings; MeSH) combined with Boolean operators. The keywords and corresponding MeSH terms used were “chronic stress,” “burnout,” “occupational burnout,” “exhaustion disorder,” “biological,” “psychosocial,” “biopsychosocial,” “biomarker,” “allostatic load,” “indicator,” “measurement,” “assessment,” “heart rate variability,” “psychometric,” “instrument,” “tool,” “survey,” “questionnaire,” “systematic review,” and “meta-analysis.” The full search strings for each database are provided in Appendix A.

### 2.4 Data collection

First, all articles retrieved from the databases (a total of 5,641 articles) were uploaded into Rayyan, an online tool for systematic reviews, and duplicates were removed, leaving 2,645 unique records. One author conducted an initial title and abstract screening. To ensure a thorough title and abstract screening, we first tested the preliminary inclusion and exclusion criteria on two separate data samples (one of 20 articles and another of 30 articles). Discrepancies were discussed between three reviewers, and the criteria were refined accordingly. After this refinement, the first author completed the title and abstract screening, resulting in 219 articles selected for full-text review. To further calibrate our refined criteria at the full-text level, we first selected a data sample of 10 full-text articles, which was independently evaluated by three reviewers. During the discussion of this sample, we introduced additional refinements, clarifying that only articles focusing explicitly on chronic stress, whether framed as chronic stress itself or in the context of burnout (including terminology such as “prolonged,” “persistent,” “long-term,” or “sustained,” stress, whether or not in combination with “(clinical) burnout,” “job burnout,” “occupational burnout” or “exhaustion disorder”), would be included. We excluded articles centered on acute or short-term stress, without clearly positioning this form of stress as chronic stress. Consequently, articles referring primarily to “work-related stress,” “workplace stressors,” “work environment,” or “working conditions,” without indicating chronicity were excluded. After integrating these further refinements, two reviewers independently screened another set of 10 full-text articles, confirming that the criteria were now consistently interpretable. With these criteria firmly in place, one reviewer proceeded to screen all remaining full-text articles. Finally, 45 articles remained. The study-selection process is depicted in Figure 1.

### 2.5 Data analysis

To synthesize the finding from the included reviews, for each review information was extracted on author, publication year, country of origin, type of review, the presence and definition or conceptualization of chronic stress, the explanatory model used, the primary outcome measures used to assess chronic stress, any secondary outcomes and the main findings and conclusions regarding the discriminative value of the assessment tools. The extracted information was then summarized and organized thematically. Results were grouped according to (1) definitions and conceptualizations of chronic stress, (2) the explanatory models (explaining the relationship between stress and health), (3) measurement approaches involving biomarkers, and (4) measurement approaches using self-reported burnout or stress scales (5) main results and (6) conclusion. This narrative synthesis allowed for comparison across studies and identification of common patterns and gaps in the assessment of chronic stress.

## 3 Results

### 3.1 Study characteristics

A total of 45 reviews were included, of which were mostly systematic reviews (*N* = 26), meta-analysis (*n* = 6) and combined reviews (*n* = 6), and seven other reviews (narrative, scoping or literature synthesis). The reviews were conducted across 18 distinct national settings most frequently the USA (*n* = 13); the Netherlands (*n* = 4); Sweden (*n* = 3); Germany (*n* = 2). Most reviews drew on general or working-adult populations, often focusing on specific occupational groups (e.g., teachers, healthcare professionals, white-collar workers), some reviews examined clinical cohorts (e.g., individuals with diagnosed burnout, major depressive disorder, Post Traumatic Stress Disorder (PTSD), diabetes, or cardiovascular disease). Some reviews selected their study populations based on exposure to specific chronic stressors, such as caregiving, unemployment, or low socioeconomic status.

In the following paragraphs, we address all four research questions. First, we will discuss the different definitions and conceptualizations of chronic stress, whether or not in the context of burnout. Secondly, the different explanatory models that are being used in the included literature are discussed, explaining the relationship between chronic stress and adverse health outcomes. Thirdly, we will discuss what measurement parameters, ranging from psychosocial indicators to physiological biomarkers, are currently used to assess chronic stress. Fourthly, we will examine the discriminative value of the different chronic stress and burnout measurements scales in differentiating healthy people from those at risk for adverse health or diagnosing (clinical) burnout.

### 3.2 Definitions of chronic stress

All the included reviews indicate that chronic stress, unlike acute stress, negatively affects health and is consistently characterized by its long-term nature. Even though not one review uses the exact same definition of chronic stress, two main categories can be distinguished. Chronic stress is either defined as (1) persistent exposure to external or internal stressors (e.g., Stressful circumstances arising from one’s social or working context, such as prolonged exposure to poverty, violence, extended caregiving or adverse working conditions, or having negative thoughts) (Segerstrom and Miller, 2004; Chida and Steptoe, 2009; Danhof-Pont et al., 2011; Wheeler et al., 2011; Grossi et al., 2015; An et al., 2016; Mathur et al., 2016; Oliveira et al., 2016; Allen et al., 2017; De Looff et al., 2018; Järvelin-Pasanen et al., 2018; Jonsdottir and Sjors Dahlman, 2019; Milaniak and Jaffee, 2019; Coimbra et al., 2020; Kaltenegger et al., 2021; Schaafsma et al., 2021; Shoman et al., 2021; Walsh et al., 2021; Finlay et al., 2022; Lindsäter et al., 2022; Michel et al., 2022; Shoman et al., 2022; Emal et al., 2023; McGee et al., 2023; Pate et al., 2023; Aguayo-Estremera et al., 2024; De Beer et al., 2024; Figueiredo et al., 2024; Hagan et al., 2024; Villacura-Herrera et al., 2025), or as (2) sustained activation of the physiological stress response (e.g., leading to heightened neural and neuroendocrine responses) (Dowd et al., 2009; Johnson et al., 2013; Wosu et al., 2013; Ottaviani et al., 2016; Bakusic et al., 2017; Johnson et al., 2017; Wiegand et al., 2017; Kim et al., 2018; Picard and McEwen, 2018; Kalliokoski et al., 2019; Guidi et al., 2021; Noushad et al., 2021; Phillips et al., 2021; Murkey et al., 2022). These two definitions of chronic stress highlight different aspects of the same phenomenon, but there is a subtle conceptual distinction between them. The first definition frames chronic stress as prolonged exposure to both external and internal stressors. In contrast, the second definition, centered on persistent activation of the stress system, emphasizes the biological reaction to a stressor, highlighting the body’s sustained physiological response to stressors as a cause of adverse stress related health outcomes.

Based on the included literature, seven interrelated dimensions of stress were identified that structure how chronic stress is currently studied in scientific literature (see Table 1): (1) explanatory models linking stress to health outcomes, (2) definitions of chronic stressors, (3) methods for measuring these stressors, (4) definitions of chronic stress reactions, (5) methods for measuring these reactions, (6) stress-related health outcomes, and (7) the assessment of these stress-related health outcomes. These seven dimensions can be found in Table 1 and are described in more detail below.

**Table 1:** Definitions and measurement approaches of chronic stress.

Explanatory models *Explaining the relationship between chronic stress and adverse health outcomes*	Chronic stressors *Possible causes of chronic stress*	Measurement of chronic stressors Assessment of chronic exposure to stressors	Chronic stress reactions *Possible biological and psychological reactions to chronic stress*	Measurement of chronic stress reactions *Mediators linking chronic stress to adverse health outcomes*	Adverse health outcomes *Adverse mental and physical health outcomes related to chronic stress*	Measurement of adverse health outcomes Measuring mental and physical health outcomes
Biopsychosocial model Dysregulation of the HPA axis and the ANS Allostatic load theory Psychosocial model Job demands-resources Job demand-control Effort-reward imbalance Organizational injustice Conservation of resources theory Burnout (Person-environment fit (transactional) model of energy depletion and available resources)	External stressors Psychosocial stressors (e.g., Early life adversity, life stress, low SES, caregiving, discrimination, loneliness, unemployment) Adverse working conditions (e.g., Job stress, night/shift work, stress related to a profession) (e.g., teachers, health care workers, military/medical training, exams) Internal stressors Perseverative cognition (ruminating thoughts about the past and worrying about the future)	External stressors SES: Income, education, childhood trauma questionnaires, cumulative ACE’s, life events checklist Caregiving, unemployment: Yes/No, durationNFR) Job stress questionnaires (e.g., ERI, JDR, OC, JCQ, NFR) Internal stressors Perseverative cognition (state perseverative cognition, trait perseverative cognition)	Biological stress reactions Physiological responses related to the neuroendocrine (HPA-axis), immune, metabolic and cardiovascular system, epigenetics, cellular aging Psychological stress reactions Perceived stress, changes in mood and emotion Behavioral stress reactions Cognitive impairments Overall cognitive ability, attention, memory Sleep disturbances Insomnia, poor sleep quality	Biological stress reactions Biomarkers related to endocrine system (e.g., cortisol), immune system (e.g., CRP, cytokines), metabolic system (e.g., cholesterol), ANS and cardiovascular system (e.g., BP, HR, HRV), cellular aging (e.g., epigenetics, telomere length and mitochondria), AL index (6–24 biomarkers, e.g., cortisol, BMI, HR, HRV, CRP, glucose) Psychological stress reactions Perceived stress scales (e.g., PSS, TICS, Perceived discrimination, loneliness) Cognitive impairments: Objective and subjective cognitive measurement tools. Sleep disturbances: Polysomnographic recordings, electronic diaries for sleep disturbances.	(Clinical) Burnout Exhaustion disorder Mental health disorders Depression Anxiety Bipolar disorder PTSD Physical health disorders CVD/Acute myocardial infarction Hypertension Diabetes II Obesity Chronic pain	(Clinical) Burnout: E.g., MBI, OLBI, BAT, SMBM, CBI, ProQOL, Single item burnout question, DSM IV Adjustment disorder, somatoform disorder, neurasthenia Exhaustion disorder: Diagnostic criteria ED, KEDS, KES, SMBM, WHODAS 2.0 Mental health disorders Depression: e.g., BDI, SCID Anxiety: e.g., STAI Bipolar disorder, PTSD: patients Yes/No Physical health disorders Diagnosis by a medical professional (patient Yes/No)

ACE, adverse childhood experiences; ACTH, adrenocorticotropic hormone; ANS, Autonomic Nervous System; BAT, Burnout Assessment Tool; BDI, Beck Depression Inventory; BMI, body mass index; BP, blood pressure; CBI, Copenhagen Burnout Inventory; CRP, C-reactive protein; CVD, cardiovascular disease; DHEAS, dehydroepiandrosterone sulfate; DSM IV, Diagnostic and Statistical Manual of Mental Disorders; ED, Exhaustion Disorder; ERI, Effort Reward Imbalance scale; GABA-A, gamma-aminobutyric-acid-A; HbA1C, glycated hemoglobin; HPA-axis, Hypothalamus-Pituitary-Adrenalin axis; HR, heart rate; HRV, heart rate variability; IL, interleukin; JCQ, Job Content Questionnaire; JDR, Job Demands Resources Scale; KEDS, Karolinska Exhaustion Disorder Scale; MBI, Maslach Burnout Inventory; NFR, Need For Recovery scale; OC, Organizational Injustice Scale; OLBI, Oldenburg Burnout Inventory; ProQOL, Professional Quality of Life; PSS, Perceived Stress Scale; PTSD, post-traumatic stress disorder; SCID, Structured Clinical Interview for DSM Disorders; SES, social economic status; SMBM, Shirom-Melamed Burnout Measurement; STAI, State–Trait Anxiety Inventory; T3, triiodothyronine; T4, thyroxine; TICS, Trier Inventory of Chronic Stress; TSH, thyroid stimulating hormone; WHODAS 2.0, World Health Organization Disability Assessment Schedule.

### 3.3 Explanatory models

**Figure 2:**
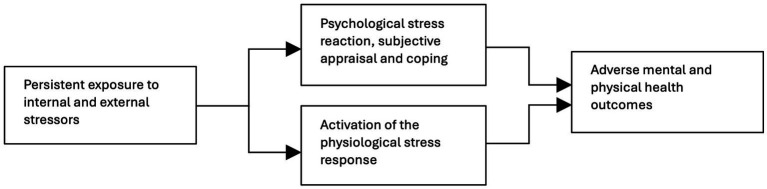
The biopsychosocial explanatory model.

The majority of the included reviews (34 out of 45) conceptualize chronic stress primarily within a biopsychosocial framework (see Figure 2), meaning that chronic stress is understood as arising from both contextual factors (external stressors such as adverse working conditions or low socioeconomic conditions), psychological variables (such as perseverative cognition, personality traits, coping strategies) and the physiological stress response (e.g., dysregulation of the HPA axis, autonomic nervous system, immune system, and metabolic pathways) (see Appendix B for an alphabetized list of all included articles, including the explanatory models each employs). The *biopsychosocial perspective* integrates these three domains in an attempt to explain how chronic stress ultimately leads to adverse mental and physical health outcomes. Twenty-eight out of these 34 reviews described the biopsychosocial perspective in terms of dysregulation of the HPA axis and/or the autonomic (ANS) nervous system, resulting from prolonged exposure to internal or external stressors. Five of the 34 included reviews adopted the allostatic load theory as an extension of this perspective, framing chronic stress as the cumulative physiological burden resulting from ongoing psychological and social stressors.

**Figure 3:**
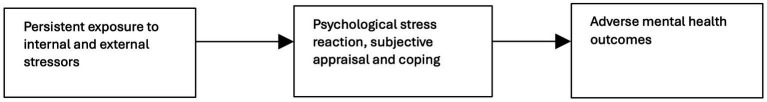
The psychosocial explanatory model.

Eleven of the included reviews approached chronic stress exclusively from a psychosocial perspective (see Figure 3), primarily focusing on burnout, conceptualized as a condition resulting from chronic occupational stress. These reviews drew on transactional models, such as the person-environment fit model which emphasize the dynamic interaction between individual perceptions and environmental demands, and the extent to which these demands can be met. Commonly applied psychosocial theories included the Conservation of Resources theory, the Job Demands-Resources theory and the Effort-Reward Imbalance theory. These perspectives highlight the role of subjective experience, coping strategies, workplace demands, and social context, rather than biological mechanisms, in the development of chronic stress.

In 18 (out of 45) of the included reviews, psychosocial and biopsychosocial perspectives were integrated to better understand the link between stress and health, particularly in occupational settings. These reviews examined associations between biological markers and burnout or work-related stress while framing occupational burnout or stress within psychosocial work stress models such as the Job Demands-Resources model, the Effort-Reward Imbalance model, and the Organizational Injustice framework. This combined approach reflects an effort to bridge socially grounded stress experiences with underlying physiological processes.

### 3.4 Measuring chronic stress

This review reveals that the included studies assess chronic stress through various approaches, whether by identifying external (psychosocial or work-related) or internal (perseverative cognition) stressors, measuring psychological and physiological stress reactions, or evaluating health outcomes associated with prolonged exposure to stress. All these forms of measurement will be discussed below.

#### 3.4.1 Measuring chronic stressors

All included reviews identified stressors, whether external or internal, as the primary drivers of chronic stress, and accordingly examined a range of psychosocial stressors in their analyses. The following section outlines the types of stressors discussed and their measurement, with over 60 distinct terms identified across reviews to describe psychosocial stressors. Many of these referred to similar underlying stressors and were therefore grouped into two broader, conceptually distinct categories to improve clarity and comparability: (1) external stressors consisting of psychosocial stressors (e.g., early life adversity, low social economic status (SES), discrimination, unemployment, caregiving) (Segerstrom and Miller, 2004; Chida and Steptoe, 2009; Dowd et al., 2009; Johnson et al., 2013; Wosu et al., 2013; An et al., 2016; Mathur et al., 2016; Oliveira et al., 2016; Allen et al., 2017; Johnson et al., 2017; Wiegand et al., 2017; Kim et al., 2018; Picard and McEwen, 2018; Kalliokoski et al., 2019; Milaniak and Jaffee, 2019; Coimbra et al., 2020; Guidi et al., 2021; Noushad et al., 2021; Phillips et al., 2021; Walsh et al., 2021; Finlay et al., 2022; Murkey et al., 2022), and adverse working conditions (e.g., job stress, shift work, working as a teacher or health care professional) (Danhof-Pont et al., 2011; Wheeler et al., 2011; Deligkaris et al., 2014; Grossi et al., 2015; Bakusic et al., 2017; De Looff et al., 2018; Järvelin-Pasanen et al., 2018; Jonsdottir and Sjors Dahlman, 2019; Kaltenegger et al., 2021; Schaafsma et al., 2021; Shoman et al., 2021; Lindsäter et al., 2022; Michel et al., 2022; Shoman et al., 2022; Emal et al., 2023; McGee et al., 2023; Pate et al., 2023; Aguayo-Estremera et al., 2024; De Beer et al., 2024; Figueiredo et al., 2024; Hagan et al., 2024; Villacura-Herrera et al., 2025), and (2) internal stressors consisting of perseverative cognition (rumination about past events or worry about future) as an internal stressor (Ottaviani et al., 2016). In Tables 1, a single label (e.g., caregiving) was used to represent multiple related stressors described across the reviews, such as caring for a spouse with dementia or a chronically ill child.

Chronic stressors were measured using a range of methods that capture both the presence of a stressor and/or the subjective experience of stress related to the stressor (e.g., perceived stress or the mental or psychological reaction to the stressor). To capture the occurrence of a stressor or stressful event, some instruments use simple dichotomous questions (yes/no) where individuals indicate whether a stressor or stressful context is present or absent (e.g., caregiving, unemployment, night/shift work). Other instruments used cumulative scores (ACE’s, stressful life events) or contextual background (e.g., measuring SES through income and education, or discrimination based on one’s background, for example being African American or Hispanic) (Chida and Steptoe, 2009; Dowd et al., 2009; Johnson et al., 2013; Mathur et al., 2016; Oliveira et al., 2016; Allen et al., 2017; Johnson et al., 2017; Wiegand et al., 2017; Kim et al., 2018; Picard and McEwen, 2018; Milaniak and Jaffee, 2019; Walsh et al., 2021; Finlay et al., 2022). Stress related to adverse working conditions was measured using work-related stress questionnaires, like the Job Demands-Control (JDC) questionnaire, Effort-Reward Imbalance (ERI), Overcommitment (OC), Need for Recovery (NFR), the Job Content Questionnaire (JCQ), the Dutch Questionnaire on the Experience and Assessment of Work (VBBA), and the Copenhagen Psychosocial Questionnaire. In most studies, researchers compare participants in the lowest (Q1) and highest (Q4) quartiles of stress exposure or symptom severity (Chida and Steptoe, 2009; Johnson et al., 2013; Bakusic et al., 2017; De Looff et al., 2018; Järvelin-Pasanen et al., 2018; Kim et al., 2018; Kaltenegger et al., 2021; Schaafsma et al., 2021; McGee et al., 2023). Perseverative cognition was assessed in two ways: through trait measures (evaluating worry or rumination) or through state measures. The latter involved experimentally inducing perseverative cognition (either directly or via induction) and subsequently measuring rumination and worry (Ottaviani et al., 2016).

Some reviews did not specify the questionnaires or methods used to measure chronic stressors (Segerstrom and Miller, 2004; Wosu et al., 2013; An et al., 2016; Kalliokoski et al., 2019; Guidi et al., 2021; Noushad et al., 2021; Phillips et al., 2021; Walsh et al., 2021; Murkey et al., 2022).

#### 3.4.2 Measuring chronic stress reactions

Chronic stress triggers a wide range of reactions in the body and mind. The included reviews all discussed the measurement of these stress reactions; to capture the effect that chronic stress has on a (1) biological, (2) psychological and (3) behavioral level we categorized the different stress reactions within these three domains.

Biological stress reactions refer to the body’s physiological responses to chronic stress. Prolonged activation of neuroendocrine pathways, such as the HPA axis and the ANS, is believed to cause widespread changes across various bodily systems, including the immune, metabolic, and cardiovascular systems. Over time, this sustained physiological dysregulation may contribute to the development of stress-related health problems. Of the 42 included reviews, 34 investigated biological stress markers. To provide clarity, we divided these biomarkers into six categories based on the physiological systems or integrative frameworks they reflect: (1) HPA-axis-related biomarkers, (2) immune system-related biomarkers, (3) metabolic system-related biomarkers, (4) biomarkers related to the ANS and the cardiovascular system, (5) biomarkers related to epigenetics and cellular aging, and (6) allostatic load indices. Each category includes a range of indicators measured using different methods depending on the biological process being assessed. Of the 42 included reviews, 34 examined physiological indicators or biomarkers of chronic stress. Nine of the 34 reviews investigated a broad range of biomarkers spanning multiple physiological systems (Danhof-Pont et al., 2011; Grossi et al., 2015; An et al., 2016; Ottaviani et al., 2016; Allen et al., 2017; Jonsdottir and Sjors Dahlman, 2019; Noushad et al., 2021; Lindsäter et al., 2022; McGee et al., 2023). These reviews exhibited considerable heterogeneity in the number of biomarkers assessed, with some studies measuring up to 59 biomarkers, while others focused on only six different indicators. Five reviews concentrated on a single biomarker either related to the HPA-axis, such as cortisol, notably hair cortisol (HCC/hCG) (Wosu et al., 2013; Kalliokoski et al., 2019; Schaafsma et al., 2021), nail cortisol (Phillips et al., 2021), and the cortisol awakening response (CAR) (Chida and Steptoe, 2009). Furthermore, five reviews specifically addressed immune-related biomarkers such as CRP, fibrinogen, IL-6, GR, and B-AR (Segerstrom and Miller, 2004; Johnson et al., 2013; Milaniak and Jaffee, 2019; Kaltenegger et al., 2021; Walsh et al., 2021). Three reviews specifically explored heart rate variability (HRV) in relation to chronic stress (De Looff et al., 2018; Järvelin-Pasanen et al., 2018; Kim et al., 2018). Seven reviews focused on emerging biomarkers related to epigenetics and cellular aging. Five of these investigated genetic and epigenetic markers, including telomere length (Mathur et al., 2016; Oliveira et al., 2016; Coimbra et al., 2020; Murkey et al., 2022) and DNA methylation (Bakusic et al., 2017), as mechanisms linking chronic stress to accelerated aging and health outcomes. Two reviews explored novel biomarkers for chronic stress, focusing on microRNAs (Wiegand et al., 2017) and mitochondrial function (Picard and McEwen, 2018). Five reviews applied an allostatic load (AL) index to assess cumulative physiological stress (Dowd et al., 2009; Johnson et al., 2017; Guidi et al., 2021; Finlay et al., 2022; Murkey et al., 2022). These indices included 6 to 24 biomarkers from endocrine, cardiovascular, immune, and metabolic systems. However, the specific biomarkers varied across reviews, highlighting inconsistencies in how AL is operationalized.

Psychological stress reactions refer to the emotional responses individuals experience in reaction to stress, these reactions are typically assessed using self-report questionnaires, such as the Perceived Stress Scale (PSS), Trier Inventory of Chronic Stress (TICS), Daily Hassles Scale, the Calgary Symptoms of Stress Inventory, and various perceived discrimination measures. (e.g., Everyday Discrimination Scale, Experiences of Discrimination (EOD)) (Chida and Steptoe, 2009; Mathur et al., 2016; Kim et al., 2018; Coimbra et al., 2020; Schaafsma et al., 2021; McGee et al., 2023).

Behavioral stress reactions refer to observable changes in functioning and daily behavior that occur in response to chronic stress, including disruptions in sleep and impairments in cognitive performance such as attention, memory, and executive functioning. Five of the included reviews examined cognitive impairments in the context of chronic stress (Deligkaris et al., 2014; Grossi et al., 2015; Allen et al., 2017; Guidi et al., 2021; Lindsäter et al., 2022). Cognitive impairments were tested objectively by submitting individuals to experimental tests in which their general cognitive ability, verbal and non-verbal memory, working memory, reaction time, attention, executive functions, and concentration were assessed. One review also asked individuals to assess their subjective cognitive performance (Lindsäter et al., 2022). Five of the included reviews looked at sleep disturbances and its association with chronic stress. Sleep disturbances were either measured through polysomnographic recordings at home, or using electronic diaries (Grossi et al., 2015; Allen et al., 2017; Guidi et al., 2021; Lindsäter et al., 2022; Michel et al., 2022).

#### 3.4.3 Measuring stress related health outcomes

Several reviews investigated the link between biomarkers and stress-related health outcomes, thereby validating whether sustained activation of the stress response plays an integral role in mediating the effects of chronic stressors on health outcomes. Because mental and physical health disorders themselves generate persistent symptoms that keep the stress system activated, they also viewed them as chronic stressors rather than merely outcomes of chronic stress, suggesting a bidirectional relation between mental and physical stress related disorders and physiological dysregulation of the stress system.

##### 3.4.3.1 Burnout

A total of thirteen reviews examined the association between biomarkers and burnout as a stress-related health outcome (Chida and Steptoe, 2009; Danhof-Pont et al., 2011; Johnson et al., 2013; Grossi et al., 2015; An et al., 2016; Bakusic et al., 2017; De Looff et al., 2018; Jonsdottir and Sjors Dahlman, 2019; Guidi et al., 2021; Noushad et al., 2021; Schaafsma et al., 2021; Lindsäter et al., 2022; McGee et al., 2023). In six of these reviews, burnout was assessed using various self-report instruments, including the MBI, SMBQ, SMBM, CBI, OLBI, and the Burnout Tedium Measure (Chida and Steptoe, 2009; Johnson et al., 2013; Bakusic et al., 2017; De Looff et al., 2018; Schaafsma et al., 2021; McGee et al., 2023). Four reviews focused specifically on clinical burnout or exhaustion disorder, aiming to identify affected individuals using a combination of self-report scales and clinical diagnostic criteria (Danhof-Pont et al., 2011; Grossi et al., 2015; Jonsdottir and Sjors Dahlman, 2019; Lindsäter et al., 2022). These reviews included studies that applied burnout scales with varying scoring methods (e.g., high/low scores, quartile comparisons, or different cut-off thresholds), alongside studies using diagnostic classifications based on DSM-IV (e.g., adjustment disorder, somatoform disorder, or neurasthenia) confirmed by healthcare professionals. Two of these reviews used formal medical diagnostic criteria for Exhaustion Disorder, recognized as a medical condition in Scandinavia, alongside instruments such as the Karolinska Exhaustion Disorder Scale (KEDS), the stress-related Exhaustion Disorder scale (s-ED), the Psychological General Well-Being Index (PGWI), and the WHO Disability Assessment Schedule 2.0 (WHODAS 2.0) (Grossi et al., 2015; Lindsäter et al., 2022). However, there were also some reviews that did not specify which instruments were used to assess burnout (Deligkaris et al., 2014; An et al., 2016; Jonsdottir and Sjors Dahlman, 2019; Guidi et al., 2021; Noushad et al., 2021).

Eleven of the included reviews approached chronic stress exclusively from a psychosocial perspective, focusing on burnout as a chronic stress related disorder stemming from persistent occupational stress that has not been well managed (Wheeler et al., 2011; Shoman et al., 2021; Michel et al., 2022; Shoman et al., 2022; Emal et al., 2023; Pate et al., 2023; Aguayo-Estremera et al., 2024; De Beer et al., 2024; Figueiredo et al., 2024; Hagan et al., 2024; Villacura-Herrera et al., 2025). The aim of these reviews was to establish the psychometric validity of various burnout measurement scales, such as the Maslach Burnout Inventory (MBI) (Wheeler et al., 2011; Aguayo-Estremera et al., 2024; De Beer et al., 2024), the Shirom-Melamed Burnout Measure (SMBM) and the Shirom-Melamed Burnout Questionnaire (SMBQ) (Michel et al., 2022; Figueiredo et al., 2024), the Burnout Assessment Tool (BAT) (Villacura-Herrera et al., 2025). Additionally, some reviews compared the relative validity of different burnout instruments, such as the Maslach Burnout Inventory (MBI), the Pine’s burnout measure (BM), the Psychologist Burnout Inventory (PBI), the Oldenburg Burnout Inventory (OLBI), the Copenhagen Burnout Inventory (CBI), the Shirom-Melamed Burnout Measure, the Burnout Assessment Tool (BAT), the Professional Quality of Life scale (ProQOL), and a single item measure (Shoman et al., 2021; Shoman et al., 2022; Pate et al., 2023). One review specifically focused on validating screening instruments for psychological distress in healthcare workers, investigating the Work functioning screener-healthcare (WFS-H), the Burnout Battery, the Physician well-being index (PWBI), the Burnout-thriving index, the Professional Quality of Life scale (ProQOL), and the Professional Fulfillment index (PFI), and a single item burnout (Emal et al., 2023).

##### 3.4.3.2 Mental health disorders

Five reviews investigated the associations between psychiatric disorders (e.g., depression, anxiety, PTSD, bipolar disorder I) and physiological biomarkers (Chida and Steptoe, 2009; Wosu et al., 2013; An et al., 2016; Bakusic et al., 2017; Picard and McEwen, 2018). When identifying a psychiatric disorder, instruments such as the Beck Depression or Beck Anxiety Inventory, Hospital anxiety and depression scale, the Anxiety and Stress scale, MDD (BDI, SSAGA, MINI, SCID) or DSM IV criteria based on a structured clinical interview were employed (Chida and Steptoe, 2009; Bakusic et al., 2017; Phillips et al., 2021). Some reviews merely noted that their participants were “patients,” without detailing which diagnostic instruments or criteria had been used to assess the psychiatric disorders (Wosu et al., 2013; An et al., 2016; Guidi et al., 2021; Noushad et al., 2021).

##### 3.4.3.3 Physical health disorders

Similarly, physical health disorders (e.g., CVD, diabetes, obesity, chronic pain) have been studied for their association with biomarkers that may mediate the link between chronic stress and disease onset and progression. Six (6) reviews investigated the association between physical health disorders (e.g., CVD, acute coronary syndrome, diabetes, adiposity, Cushing’s syndrome, Musculoskeletal disorders, chronic fatigue syndrome) and biomarkers, only mentioning whether individuals were patients, but not reporting which measurement instruments were used to assess these conditions (Wosu et al., 2013; An et al., 2016; Kalliokoski et al., 2019; Guidi et al., 2021; Noushad et al., 2021; Phillips et al., 2021).

### 3.5 Discriminative value

In this paragraph we will be answering the fourth question of this scoping review: To what extent do current chronic stress measurement tools demonstrate discriminative value in distinguishing healthy populations from those at risk for adverse health outcomes? And are these measurement tools suitable for diagnosing (clinical) burnout specifically? Below and in Tables 2–4 these results are presented.

**Table 2:** Discriminative Value of Biomarkers.

Biomarkers	Discriminative value	References
Biomarkers related to the HPA-axis.	Cortisol showed both blunted responses, elevated levels, and non-significant associations with chronic stress, depending on the nature and chronicity of the stress exposure. While nine (9) reviews concluded cortisol to be a valid biomarker, signaling hypercortisolism and hypocortisolism, particularly in large-scale studies or related to short-term ongoing stress, five (5) reviews questioned its clinical utility and diagnostic accuracy, especially in relation to clinical burnout, exhaustion disorder and occupational stress.	Chida and Steptoe (2009), Danhof-Pont et al. (2011), Wosu et al. (2013), Grossi et al. (2015), An et al. (2016), Ottaviani et al. (2016), Allen et al. (2017), Jonsdottir and Sjors Dahlman (2019), Kalliokoski et al. (2019), Noushad et al. (2021), Phillips et al. (2021), Schaafsma et al. (2021), Lindsäter et al. (2022), and McGee et al. (2023)
Biomarkers related to the metabolic system.	Findings on metabolic biomarkers were mixed and largely inconclusive, with no review supporting their use as reliable indicators of chronic stress.	Danhof-Pont et al. (2011), Grossi et al. (2015), Allen et al. (2017), and Noushad et al. (2021)
Biomarkers related to the immune system.	Eight (out of 11) reviews demonstrated a significant relationship between different stressors and low-grade inflammation as measured by elevated C-reactive protein (CRP) levels, suppression of global immunity, and increased pro-inflammatory activity and less effective anti-inflammatory activity. Even so three (3) reviews concluded that findings related to burnout were mixed and require further research.	Segerstrom and Miller (2004), Danhof-Pont et al. (2011), Johnson et al. (2013), Grossi et al. (2015), Allen et al. (2017), Jonsdottir and Sjors Dahlman (2019), Milaniak and Jaffee (2019), Kaltenegger et al. (2021), Noushad et al. (2021), Walsh et al. (2021), and McGee et al. (2023)
Biomarkers related to the ANS and the cardiovascular system.	Five (5) out of nine (9) reviews found that chronic stress, particularly related to work and perseverative cognition, is associated with reduced heart rate variability (HRV) and elevated heart rate and blood pressure. Four (4) reviews concluded that results were inconsistent across stressor types and conditions such as burnout, highlighting the need to interpret cardiovascular biomarkers like HRV in the context of individual psychological and medical factors.	Danhof-Pont et al. (2011), Grossi et al. (2015), Ottaviani et al. (2016), Allen et al. (2017), De Looff et al. (2018), Järvelin-Pasanen et al. (2018), Kim et al. (2018), Lindsäter et al. (2022), and McGee et al. (2023)
Telomere length	Four (4) reviews found that chronic stress was associated with shorter telomere length.	Mathur et al. (2016), Oliveira et al. (2016), Coimbra et al. (2020), and Murkey et al. (2022)
DNA methylation	One (1) review found that chronic stress was associated with increased DNA methylation in HPA axis-related genes.	Bakusic et al. (2017)
miRNAs	One (1) review found altered expression of specific micro ribonucleic acid (miRNAs) in response to chronic stress.	Wiegand et al. (2017)
Mitochondria	One (1) review linked different chronic stressors to maladaptive mitochondrial changes affecting energy production.	Picard and McEwen (2018)
Allostatic Load-index*	Five (5) reviews concluded that higher allostatic load was associated with chronic stress.	Dowd et al. (2009), Johnson et al. (2017), Guidi et al. (2021); Finlay et al. (2022), and Murkey et al. (2022)

*Allostatic Load index: Mix of 6–25 neuroendocrine, cardiovascular, metabolic and immune biomarkers including cortisol, waist hip ratio, Body Mass Index, Interleukin, Fibrinogen, CRP (C-Reactive Protein), Creatine, IG-F (Insulin-like growth factor).

**Table 3:** Discriminative value of sleep disturbances and cognitive impairments.

Symptoms	Discriminative value	References
Sleep disturbances	Sleep disturbances, particularly insomnia-type impairments, were consistently linked to clinical burnout, exhaustion disorder, and high allostatic load; they appear to function as both causative and maintaining factors and were more prevalent in at-risk groups such as caregivers.	Grossi et al. (2015), Allen et al. (2017), Guidi et al. (2021), Lindsäter et al. (2022), and Michel et al. (2022)
Cognitive impairments	Cognitive impairments, particularly in executive function, attention, and memory, were associated with clinical burnout, exhaustion disorder, caregiving, and high allostatic load.	Deligkaris et al. (2014), Grossi et al. (2015), Allen et al. (2017), Guidi et al. (2021), and Lindsäter et al. (2022)

**Table 4:** Discriminative value of biomarkers and burnout measurement scales in diagnosing (clinical) burnout and exhaustion disorder.

Biomarkers and burnout measurement scales	Stress related mental health outcome	Discriminative value	References
Different biomarkers of the neuroendocrine, immune, metabolic and cardiovascular system.	(Clinical) burnout exhaustion disorder	No biomarker was found to be suitable for diagnostic purposes in relation to (clinical) burnout or exhaustion disorder.	Danhof-Pont et al. (2011), Grossi et al. (2015), Jonsdottir and Sjors Dahlman (2019), and Lindsäter et al. (2022)
KEDS SMBQ-22 PGWI WHODAS 2.0	Exhaustion disorder	The KEDS and the SMBQ were found to discriminate between those who have received an exhaustion disorder diagnosis and heathy individuals, even so further validation is required regarding their ability to differentiate Exhaustion Disorder (ED) from other conditions.	Lindsäter et al. (2022)
MBI	Burnout	Although the EE dimension showed the strongest reliability, none of the subscales of the MBI were found to meet the rigorous standard for diagnostic accuracy.	Wheeler et al. (2011), Aguayo-Estremera et al. (2024), and De Beer et al. (2024)
MBI BM PBI OLBI CBI SMBM SMBQ BAT ProQOL Single Item Burnout Question	Burnout	The BAT, and to a lesser extent the SMBM, were found to have acceptable psychometric properties but should not be used for clinical purposes.	Shoman et al. (2021), Michel et al. (2022), Shoman et al. (2022), Pate et al. (2023), Figueiredo et al. (2024), Hagan et al. (2024), and Villacura-Herrera et al. (2025)
WFS-H Burnout Battery PWBI ProQOL Burnout Thriving Index Single-item burnout PFI	Psychological distress	Existing screening tools for psychological distress in healthcare workers were found to show low methodological quality and insufficient evidence for diagnostic accuracy.	Emal et al. (2023)

EE, emotional exhaustion; KEDS, Karolinska Exhaustion Disorder Scale; SMBQ (−22), Shirom-Melamed Burnout Questionnaire; PGWI, General Well-Being Index; WHODAS 2.0, World Health Organization Disability Assessment Schedule 2.0; MBI, Maslach Burnout Inventory; BM, Pine’s Burnout Measure; PBI, Psychologist Burnout Inventory; OLBI, Oldenburg Burnout Inventory; CBI, Copenhagen Burnout Inventory; SMBM, Shirom-Melamed Burnout Measure; BAT, Burnout Assessment Tool; Work-functioning screener-healthcare (WFS-H); PSS, Perceived Stress Scale; WFS-H, work functioning screener-healthcare; PWBI, Physician Wellbeing Index; ProQOL, Professional Quality of Life; PFI, Professional fulfillment index.

#### 3.5.1 Discriminative value of biomarkers in assessing chronic stress

Thirty-four of the included reviews explore the validity of the biopsychosocial perspective. They aim to investigate stress health relationships by integrating biological mechanisms (e.g., HPA-axis activation, neuroendocrine responses) with psychosocial stressors (e.g., worrying, ruminating, caregiving socioeconomic strain, occupational stress) and stress related physical and mental health disorders. By linking biomarkers to both various chronic stressors and adverse stress related health outcomes (e.g., burnout, depression, cardiovascular disease, and diabetes), researchers tried to gain deeper insight into how prolonged stress takes a toll on the body and contributes to the development of stress-related conditions. Results are presented in Table 2.

##### 3.5.1.1 Biomarkers related to the HPA-axis

Fourteen reviews investigated cortisol as a biomarker, with some focusing exclusively on it. As a key HPA-axis hormone involved in the stress response, cortisol is a common target in chronic stress research. However, findings on its association with various chronic stressors and stress-related health outcomes were mixed across reviews. While 9 reviews conclude that cortisol is a promising indicator of chronic stress (Chida and Steptoe, 2009; Wosu et al., 2013; An et al., 2016; Ottaviani et al., 2016; Allen et al., 2017; Kalliokoski et al., 2019; Noushad et al., 2021; Phillips et al., 2021; McGee et al., 2023), five found the results too inconsistent to support this view (Danhof-Pont et al., 2011; Grossi et al., 2015; Jonsdottir and Sjors Dahlman, 2019; Schaafsma et al., 2021; Lindsäter et al., 2022). Reviews supporting the use of cortisol, suggest that chronic stress can manifest in different ways, with studies reporting both blunted and elevated cortisol responses. Even though the effects might seem different, and results vary between types of stressors and ways of measuring cortisol, these reviews conclude that chronic stress can be related to dysregulation of the HPA axis (whether it be an upregulation, or a downregulation), suggesting the validity of cortisol and making it a potential biomarker in measuring chronic stress as a prognostic instrument.

However, five reviews investigating the relation between cortisol and clinical burnout, exhaustion disorder, or occupational stress, found no consistent association with cortisol levels (Danhof-Pont et al., 2011; Grossi et al., 2015; Jonsdottir and Sjors Dahlman, 2019; Schaafsma et al., 2021; Lindsäter et al., 2022). Consequently, these authors determined that cortisol cannot serve as a reliable diagnostic biomarker. They attributed this to inconsistencies in the definition and measurement of burnout, including varying cutoff scores for the MBI and SMBQ, the use of self-report questionnaires, and inconsistent assessment methods for biomarkers. While Grossi et al. (2015) acknowledged the theoretical plausibility of both hyper- and hypocortisolism in burnout, Jonsdottir and Sjors Dahlman (2019) argued that HPA-axis activity is a useful marker for acute stress reactions but may be less suitable for chronic stress.

##### 3.5.1.2 Biomarkers related to the metabolic system

Danhof-Pont et al. (2011) investigated metabolic biomarkers such as cholesterol, glucose, HbA1c (glycated hemoglobin), triglycerides, BMI (body-mass-index), and insulin, but did not report on their outcomes. Other reviews showed mixed results: Grossi et al. (2015) and Allen et al. (2017) found inconsistent associations between caregiving or burnout and markers like HbA1c, glucose, insulin, and BMI, with Allen et al. (2017) suggesting stronger effects when distress is more substantial. Noushad et al. (2021) reported some findings linking high chronic stress to elevated glucose, HbA1c, and cholesterol, and noted poorer glucose regulation in Hispanic individuals prior to diabetes onset. Overall results showed to be fragmented or non-significant, with no review concluding that metabolic markers could be used as potential indicators of chronic stress or even referring to associations between metabolic markers and chronic stress in the concluding section of the review.

##### 3.5.1.3 Biomarkers related to the immune system

Eleven reviews investigated immune function in relation to chronic stress. Eight reviews found consistent data linking chronic stressors to immune dysregulation. Various psychosocial stressors such as caregiving, living with a disability, unemployment and (teacher) burnout and job stress, low socioeconomic status, and discrimination were found to correlate with suppressed global immunity, decreased immune function, a decrease in anti-inflammatory cytokines, a higher overall pro-inflammatory activity, low-grade inflammation (indicated by elevated CRP levels) (Segerstrom and Miller, 2004; Johnson et al., 2013; Noushad et al., 2021; McGee et al., 2023). Furthermore, evidence was found that immune activity in response to challenges was altered in caregivers, causing delayed recovery (in wound healing) and more days with infectious illness (Allen et al., 2017). Early childhood socioeconomic status was found to predict chronic inflammation markers early in life (Milaniak and Jaffee, 2019), and chronic social stress tied to caregiving, social isolation, and bereavement was similarly linked to increased peripheral inflammatory proteins (Walsh et al., 2021). While Kaltenegger et al. (2021) found no direct associations between work stress and immune markers, they observed that workplace interventions emphasizing physical activity reduced CRP levels, suggesting its usefulness in monitoring chronic stress. Overall, these findings underscore the central role of immune markers in reflecting the physiological impact of chronic stress.

In relation to burnout and exhaustion disorder, immune markers showed mixed findings (Danhof-Pont et al., 2011; Grossi et al., 2015; Jonsdottir and Sjors Dahlman, 2019; Lindsäter et al., 2022), with researchers concluding that immune markers could not serve as a diagnostic biomarker. Although some differences between patients and controls reached statistical significance, most values remained within normal clinical ranges. Jonsdottir and Sjors Dahlman (2019) emphasized the importance of studying more homogeneous populations, as physiological responses may vary between working individuals and clinically diagnosed groups experiencing severe symptoms and sickness absence.

##### 3.5.1.4 Biomarkers related to the ANS and the cardiovascular system

Five reviews reported that occupational chronic stress (burnout), job stress, and perceived stress and perseverative cognition (worrying about the future and ruminating about the future) were linked to increased heart rate, elevated blood pressure, and lowered HRV, reflecting reduced parasympathetic activity (Ottaviani et al., 2016; De Looff et al., 2018; Järvelin-Pasanen et al., 2018; Kim et al., 2018; McGee et al., 2023). Four other reviews found mixed results on HRV in relation to caregiving, burnout and exhaustion disorder (Danhof-Pont et al., 2011; Grossi et al., 2015; Allen et al., 2017; Lindsäter et al., 2022), concluding that the broad range of variables assessed using multiple methodologies likely have contributed to these inconsistent results. Although most reviews suggest cardiovascular indicators, especially HRV, may serve as an objective marker of chronic stress, the authors emphasize that HRV should be interpreted in the context of an individual’s psychological and medical history, given that HRV reflects overall autonomic health rather than a specific mental illness.

##### 3.5.1.5 Biomarkers related to epigenetics and cellular aging

Four reviews examined the association between chronic stress and telomere length, with the majority providing support for telomere shortening as a consequence of chronic stress exposure. Several psychosocial stressors, including caregiving, low socioeconomic status, childhood adversity, racial discrimination, and cumulative life stress, consistently showed strong associations with telomere shortening (Oliveira et al., 2016; Murkey et al., 2022). Although perceived stress was associated with shorter telomeres, the reported effect size was small, suggesting telomere length may more accurately reflect long-term rather than short-term stress (Mathur et al., 2016). Findings regarding social discrimination, however, were less consistent: Coimbra et al. (2020) reported only weak associations. Overall, despite promising findings supporting telomere length as a biomarker of chronic stress, additional longitudinal research is required to confirm its clinical relevance.

Bakusic et al. (2017) reported increased DNA methylation mechanisms in HPA-axis related genes in stress, burnout, work stress, and depression. Although specific genes such as NR3C1, SLC6A4, BDNF, and TH exhibit different methylation patterns in response to various chronic stressors, these findings shed light on the complex mechanisms driving stress-related health disorders. Further research is needed to validate methylation as a biomarker for chronic stress. Meanwhile, Wiegand et al. (2017) observed that salivary miRNAs may serve as a noninvasive biomarker in the context of academic exam stress, though more work is required to confirm their diagnostic potential.

One review investigated mitochondrial function, an emerging biomarker associated with cellular aging, as a potential indicator of chronic stress. Overall, findings suggests that chronic stressors can significantly alter mitochondrial functioning, notably affecting the cells’ energy production capacity. While animal studies have provided robust evidence supporting this link, human studies remain limited and are primarily observational (Picard and McEwen, 2018). Consequently, the authors emphasize that, in humans, the assessment of chronic stress should not rely solely on biomarkers but should also include functional measures.

##### 3.5.1.6 Allostatic load index and its associations with chronic stress

Five reviews studied the Allostatic Load Index (AL-index), which combines multiple biomarkers across different biological systems, typically including cardiovascular, metabolic, neuroendocrine (e.g., HPA-axis), inflammatory, and immune indicators. These reviews found that lower socioeconomic status (SES) was consistently associated with elevated allostatic load (AL), particularly through cardiovascular and metabolic biomarkers, while endocrine measures showed weaker associations (Dowd et al., 2009; Johnson et al., 2017). Elevated AL was also linked to adverse health outcomes related to work-related stress, unhealthy lifestyle habits, and childhood adversity, highlighting the long-term physical and psychological impact of chronic stress (Guidi et al., 2021; Finlay et al., 2022). Furthermore, higher chronic stress combined with lower income was specifically correlated with increased AL among African Americans (Murkey et al., 2022). Although the AL-index appears promising as an early detection tool, the authors emphasize the need for a standardized “gold standard” and recommend supplementing biological markers with clinimetric criteria to better capture individual-level variation.

#### 3.5.2 Discriminative value of cognitive function and sleep disturbances

Sleep disturbances and cognitive impairments are recognized symptoms of chronic stress. Several reviews examined the extent to which these symptoms are characteristic of prolonged exposure to psychosocial stressors or stress related health conditions. The results are presented in Table 3.

Sleep disturbances were found to be significantly related to clinical burnout, burnout measured with the SMBM, and exhaustion disorder functioning both as a causative and maintaining factor (Grossi et al., 2015; Lindsäter et al., 2022; Michel et al., 2022), with one review stating sleep disturbances as a significant predictor of ED (Grossi et al., 2015). Poor sleep quality was also linked to high allostatic load and identified as both a consequence and a component of broader health-damaging behaviors (Guidi et al., 2021). Furthermore, caregivers, particularly males caring for spouses with severe dementia, showed reduced percentage of sleep, increased time in non-restorative sleep stages (N1 and R), earlier wake after sleep onset, and poorer sleep efficiency (Allen et al., 2017).

Reviews investigating cognitive impairments in the context of chronic stress found that individuals with (clinical) burnout or exhaustion disorder typically showed deficits in executive functions, attention, and episodic and working memory (Deligkaris et al., 2014; Grossi et al., 2015; Lindsäter et al., 2022). Although one review showed only mild cognitive impairments on psychometric tests in relation to exhaustion disorder, subjective reports of cognitive deficits were consistently more pronounced compared to controls (Lindsäter et al., 2022). Caregivers were found to show poorer performance on tests of attention and executive function, while findings on memory were mixed, with some studies reporting impairments and others not (Allen et al., 2017). High allostatic load was found to be associated with cognitive decline and structural brain changes, particularly in older adults and those with psychiatric conditions (Guidi et al., 2021).

#### 3.5.3 Discriminative value of biomarkers and burnout measurement scales in diagnosing (clinical) burnout and exhaustion disorder

This section presents the findings from the included literature regarding the diagnosis of (clinical) burnout and exhaustion disorder. While four reviews explored both potential biomarkers and psychosocial scales for diagnostic purposes, eleven reviews focused exclusively on evaluating the psychometric properties of various self-rated burnout questionnaires. The results are presented in Table 4.

Four reviews assessed physiological stress biomarkers as potential diagnostic or predictive indicators for clinical burnout or Exhaustion Disorder (ED). Despite examining clinically diagnosed cases, all concluded that the biomarkers were too inconsistent and unreliable to serve as diagnostic tools (Danhof-Pont et al., 2011; Grossi et al., 2015; Jonsdottir and Sjors Dahlman, 2019; Lindsäter et al., 2022).

Twelve reviews assessed the psychometric quality of different psychosocial self-rated measurements scales to determine burnout and exhaustion disorder as an adverse health outcome of chronic stress. One review specifically investigated the psychometric properties of scales used in diagnosing Exhaustion Disorder, concluding that the Karolinska Exhaustion Disorder Scale (KEDS) was able to discriminate individuals with ED from healthy controls with a sensitivity and specificity above >95%, while the Shirom-Melamed Burnout Questionnaire (SMBQ) also performed well with values over 83%; however, they concluded that further validation is required regarding their ability to differentiate Exhaustion Disorder (ED) from other conditions (Lindsäter et al., 2022). Three other reviews investigated the psychometric properties of the Maslach Burnout Inventory (MBI), and concluded that, although widely used, the MBI, despite good reliability of the Emotional Exhaustion (EE) dimension (0.88), did not meet the stringent diagnostic accuracy criteria necessary for clinical decision-making (0.90 minimum, 0.95 ideal), and thus was not recommended for diagnostic purposes (Wheeler et al., 2011; Aguayo-Estremera et al., 2024; De Beer et al., 2024). Two reviews specifically investigated the validation of the Shirom-Melamed Burnout Measure (SMBM) and the Shirom-Melamed Burnout Questionnaire (SMBQ), and concluded that, although the questionnaires were well grounded in the Conservation of Resources theory (COR) (Hobfoll, 1989, 2002) and positively related to the JD-R framework, it was only moderately predictive of job burnout, and advised to only be used in research practice and not as a diagnostic tool (Michel et al., 2022; Figueiredo et al., 2024). A meta-analysis by Villacura-Herrera et al. (2025) concluded that the Burnout Assessment Tool (BAT) demonstrated high reliability for assessing employee wellbeing in organizational contexts. However, its application in diagnostic settings should be approached with caution due to the higher precision such contexts demand. A meta-analysis found that the SIBOQ can help identify different levels of burnout within an organization, aiding organizations in identifying areas of strength and concern. However, its ability to measure burnout accurately is limited when results need to be compared to other organizations. A systematic review of the Single Item Burnout Questionnaire (SIBOQ) concluded that, although it showed adequate reliability compared to the Emotional Exhaustion (EE) scale of the MBI, it cannot be recommended as a brief and reliable measure of burnout for use in large workplace studies due to its limited ability to provide accurate results when comparing across organizations (Hagan et al., 2024). Additionally, two other reviews by Shoman et al. (2021) and Shoman et al. (2022) investigated the psychometric properties of different burnout measurement scales (MBI, BM, PBI, OLBI, CBI, SMBM and BAT), concluding that although the Burnout Assessment Tool (BAT) had the best psychometric validity, the quality of evidence for some properties was low or very low leaving much to be desired in discriminating burnout from other affective states, suggesting a need for additional validation studies. Two reviews summarized different validated burnout measurement tools, with one listing 11 most cited validated reliable surveys (Pate et al., 2023), and one specifically assessing the diagnostic accuracy of instruments designed to measure psychological distress in healthcare workers (Emal et al., 2023). Both reviews concluded that due to methodological limitations, it remained unclear whether these measurement tools could reliably distinguish healthy individuals from those at risk of burnout or psychological distress.

## 4 Discussion

There is currently no universally accepted definition of burnout, nor are there standardized diagnostic criteria. This means that burnout lacks formal recognition as a clinical diagnosis. Although burnout is officially defined as “the result of chronic workplace stress that has not been successfully managed” by the World Health Organization (2019) and World Health Organization (2022), ICD-11, this broad framing fails to reflect the complexity of clinical burnout, which involves severe symptoms, long-term functional impairment, and extended absence from work and daily activities (Van Dam, 2021; Schaufeli et al., 2023a). Given that clinical burnout is widely considered a consequence of prolonged exposure to chronic stress, a critical first step in advancing diagnostic clarity is to understand how chronic stress is currently defined and measured in the literature. This integrative scoping review therefore aimed to (1) map current conceptualizations and definitions of chronic stress, (2) identify the explanatory models currently used to describe how chronic stress influences health, (3) categorize different assessment methods of chronic stress, and (4) evaluate the discriminative value of these tools for identifying individuals at risk for chronic stress and in diagnosing clinical burnout.

This scoping review synthesized findings from 45 (systematic) reviews and meta-analysis, encompassing over 2,000 individual studies. Together, these sources reflect a vast and diverse body of research on chronic stress and burnout.

In examining how chronic stress is defined in the literature (research question nr. 1), we found that it is a heterogeneous concept. Across reviews, chronic stress was variously used to describe the prolonged presence of stressors, the sustained biological and psychological responses they evoke, or the resulting mental and physical adverse health outcomes to which they contribute, including (clinical) burnout. Even so two main definitional approaches emerged: chronic stress as a long-lasting or recurring exposure to external (e.g., low SES, adverse working conditions, caregiving) or internal (e.g., ruminating, worrying) ‘stressors’, versus chronic stress as persistent physiological ‘stress reactions’ (e.g., dysregulation of the HPA-axis). Despite consensus on “duration” being critical to the concept of chronic stress, none of the reviews specified an exact timeframe.

In examining which explanatory models were employed to describe the relationship between chronic stress and health outcomes (research question nr. 2), two frameworks emerged: the biopsychosocial and the psychosocial model. The biopsychosocial model was the most frequently applied explanatory framework (used in 34 out of 45 reviews). Particularly studies investigating chronic stress conceptualized through a broad range of stressors, such as caregiving, work-related stress, burnout, socioeconomic adversity, discrimination, and unemployment, reflected on chronic stress as a multifactorial phenomenon involving biological, psychological, and social mechanisms. In contrast, the psychosocial model was mostly applied in studies focusing on work-related stress or burnout, emphasizing emotional and psychological processes while largely omitting biological components.

The measurement of chronic stress proved highly variable across studies (research question nr. 3). This review identified over 60 different conceptualizations of chronic stressors, more than 70 different physiological biomarkers used to assess biological stress responses, a wide range of psychosocial instruments assessing perceived stress, and over 20 different burnout scales. This overwhelming heterogeneity demonstrates not only a lack of consensus on the definition and measurement of chronic stress but also points to a more fundamental gap: a notable omission of conceptual discussion. None of the included reviews addressed the importance of clarifying what chronic stress is. This conceptual ambiguity is problematic for research and clinical practice alike, as it hampers comparability across studies, complicates the identification of individuals at risk, and risks undermining the development of effective interventions. Given the well-documented role of chronic stress in the onset and progression of mental and physical disorders, including clinical burnout, there is an urgent need for a unified definition and conceptual framework of chronic stress in humans.

In evaluating the discriminative value of the various measurement approaches (research question nr 4), we found that no single biomarker of burnout scale can reliably diagnose clinical burnout. When assessing the discriminative value of physiological biomarkers, none of the included reviews demonstrated sufficient accuracy to diagnose clinical burnout or exhaustion disorder (Danhof-Pont et al., 2011; Grossi et al., 2015; Jonsdottir and Sjors Dahlman, 2019; Lindsäter et al., 2022). This mirrors broader psychiatric research, where efforts to identify diagnostic biomarkers for heterogeneous conditions like major depressive disorder have also been unsuccessful (Winter et al., 2024). In addition, none of the reviewed (self-rated) burnout scales demonstrated sufficient validity to serve as a standalone diagnostic tool for diagnosing burnout (Wheeler et al., 2011; Shoman et al., 2021; Lindsäter et al., 2022; Michel et al., 2022; Shoman et al., 2022; Emal et al., 2023; Pate et al., 2023; Aguayo-Estremera et al., 2024; De Beer et al., 2024; Figueiredo et al., 2024; Hagan et al., 2024; Villacura-Herrera et al., 2025). Although the BAT, and to a lesser extend the KEDS and the SMBQ, showed ability to distinguish individuals at risk of burnout or exhaustion disorder from healthy controls, they failed to meet the threshold for criterion validity and therefore could not reliably differentiate burnout from other psychiatric disorders, highlighting the absence of a singular scale for diagnosing (clinical) burnout (Shoman et al., 2021; Lindsäter et al., 2022; Michel et al., 2022; Shoman et al., 2022). This aligns with a recent study by Schaufeli et al. (2023a) who found that while the BAT demonstrates sufficient sensitivity, specificity, and internal consistency for screening purposes, its performance falls short of the ≥0.90 threshold for clinical diagnosis and should be interpreted with caution in contexts requiring higher measurement precision. They partly attribute this limitation to heterogeneity of burnout cases and recommend using current cut-offs only provisionally until more rigorous diagnostic-accuracy studies are completed. Surprisingly, the Maslach Burnout Inventory (MBI), regarded as the ‘gold standard’ in burnout research, also proved insufficient for diagnosing (clinical) burnout. Despite its widespread use and the fact that many definitions of burnout are based on Maslach’s three-component model, the MBI’s subscales do not correlate strongly enough to support burnout as a single, unified syndrome (De Beer et al., 2024). A conclusion also emphasized by Bianchi et al. (2024), who argue that the MBI fails to measure burnout as a cohesive construct and cannot be used as a diagnostic tool.

Based on the findings of these reviews, it seems reasonable to conclude that self-rated burnout questionnaires are best used for supportive screening and should be supplemented with clinical interviews and diagnostic frameworks. A conclusion supported by Van Dam (2021) and Schaufeli et al. (2023b), who argue that, rather than relying solely on questionnaires, clinicians should reconstruct the pathogenesis by tracing key life events, symptom development, and mechanisms underlying chronic stress-related clinical burnout, using diagnostic criteria grounded in established guidelines (Schaufeli et al., 2023b).

### 4.1 Support for the biopsychosocial model of chronic stress

Although no single biomarker or self-rated psychosocial scale was able to diagnose clinical burnout definitively, the included reviews provided strong support for the biopsychosocial model of chronic stress. Across reviews, consistent associations with biological indicators validated the view of stress as a multifactorial phenomenon arising from dynamic interactions between biological, psychological, and social factors. Even though mixed results were found in relation to HPA-axis activity, both elevated and reduced cortisol levels were thought to reflect adaptive variability based on stress duration and chronicity (Chida and Steptoe, 2009; Wosu et al., 2013; An et al., 2016; Ottaviani et al., 2016; Allen et al., 2017; Kalliokoski et al., 2019; Noushad et al., 2021; Phillips et al., 2021; McGee et al., 2023), underlining the idea that both hypercortisolism and hypocortisolism can be potentially pathogenic (Miller et al., 2007). Immune system biomarkers, particularly inflammatory markers, showed consistency in correlation with chronic stress exposure (Segerstrom and Miller, 2004; Johnson et al., 2013; Allen et al., 2017; Milaniak and Jaffee, 2019; Kaltenegger et al., 2021; Noushad et al., 2021; Walsh et al., 2021; McGee et al., 2023). Cardiovascular biomarkers and indicators related to ANS activity, especially reduced heart rate variability pointing to reduced parasympathetic activation, also emerged as reliable markers of chronic stress (Ottaviani et al., 2016; Allen et al., 2017; De Looff et al., 2018; Järvelin-Pasanen et al., 2018; Kim et al., 2018; McGee et al., 2023). These consistent associations between immune and cardiovascular markers and chronic stress likely reflect their role as downstream indicators of prolonged stress exposure. A pattern which is acknowledged by the allostatic load framework, which conceptualizes the physiological effects of chronic stress as a cumulative cascade, beginning with primary mediators (e.g., cortisol), progressing to secondary system changes (e.g., immune and cardiovascular dysregulation), and ultimately resulting in disease (e.g., burnout, CVD, diabetes). Included reviews that focused on employing the allostatic load index to capture this multi-systemic burden, reinforced its utility as an integrative measure aligned with the biopsychosocial model, finding consistent associations between high allostatic load and chronic stress (Dowd et al., 2009; Johnson et al., 2017; Guidi et al., 2021; Finlay et al., 2022; Murkey et al., 2022). These findings are consistent with work by Juster et al. (2010) and Bobba-Alves et al. (2022), who emphasized how chronic stress progresses from adaptive responses to allostatic overload, ultimately driving dysregulation, disease, and accelerated aging. Additionally, novel biomarkers such as telomere shortening, mitochondrial dysfunction, and epigenetic changes further demonstrated the cellular and molecular imprint of chronic stress (Mathur et al., 2016; Oliveira et al., 2016; Bakusic et al., 2017; Wiegand et al., 2017; Picard and McEwen, 2018; Coimbra et al., 2020; Murkey et al., 2022).

**Figure 4:**
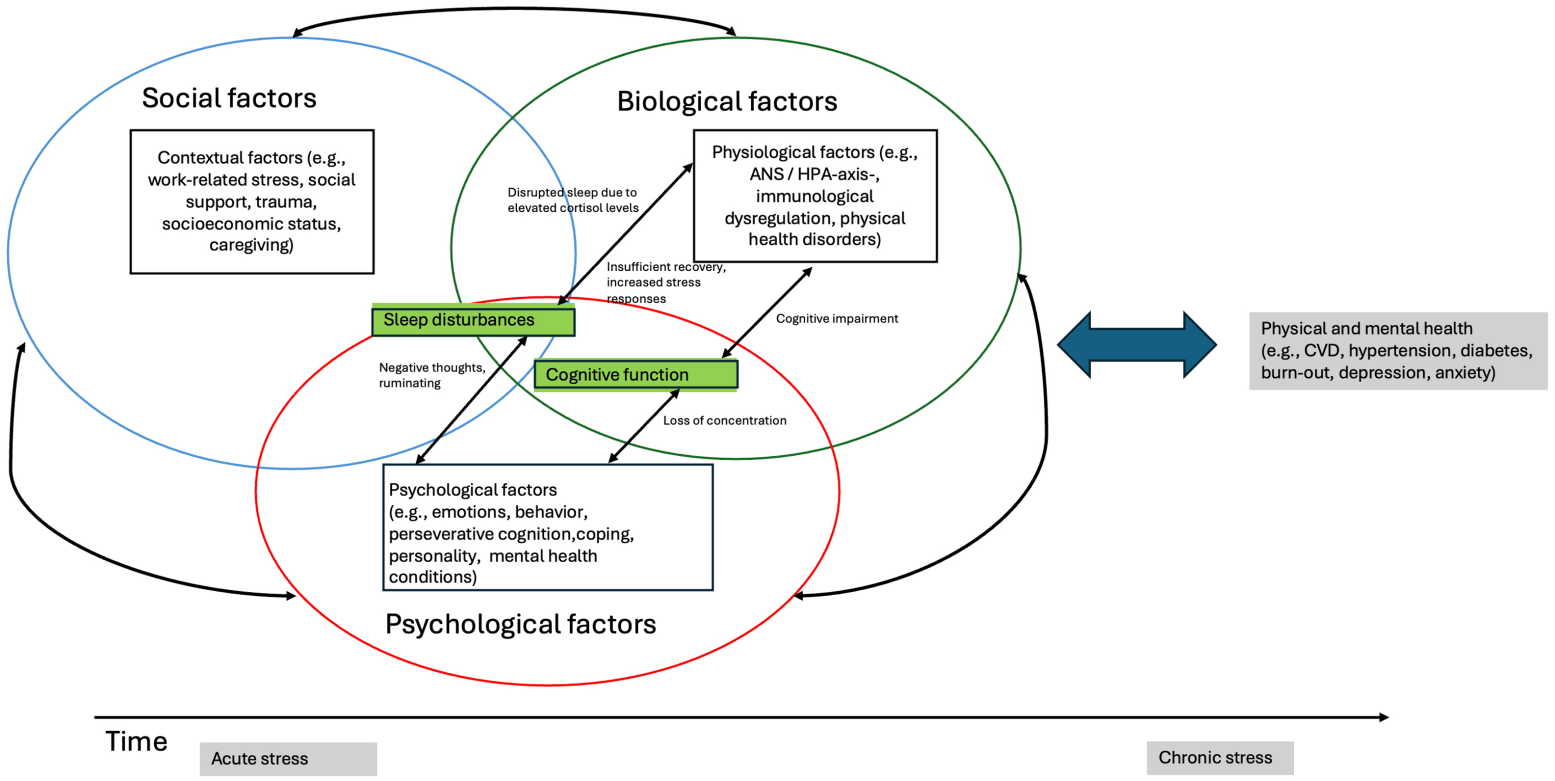
The biopsychosocial model of chronic stress.

Together, the reviewed literature strongly validates the biopsychosocial model of chronic stress (Figure 4) by illustrating how chronic stress exerts effects not only across psychological and social processes but also across interconnected biological systems, ultimately impacting both physical and mental health. Reviews also tied chronic stress to diverse physical and mental disorders, indicating bidirectional links. While chronic stress can drive the onset and progression of disorders such as depression or cardiovascular disease, yet living with these conditions likewise perpetuates psychological rumination and biological stress responses, thereby sustaining a cycle of chronic stress. Time seems to be a critical factor in this process, as stress becomes detrimental to health only when stressors and stress responses are repeated and sustained over time, gradually overwhelming the body’s adaptive capacity.

### 4.2 Sleep disturbances and cognitive impairments as key characteristics

Reviews examining the biopsychosocial model of chronic stress frequently included sleep disturbances and cognitive impairments as key characteristics, highlighting their relevance across both biological and psychological dimensions. Sleep problems, particularly insomnia-type disturbances, were shown to function as both a causative and maintaining factor of stress related complaints (Grossi et al., 2015; Allen et al., 2017; Guidi et al., 2021; Lindsäter et al., 2022; Michel et al., 2022), while cognitive impairments, especially in executive function, attention, and memory, were associated with chronic stress in a bidirectional manner (Deligkaris et al., 2014; Grossi et al., 2015; Allen et al., 2017; Guidi et al., 2021; Lindsäter et al., 2022). These findings highlight the potential relevance of sleep disturbances and cognitive impairments in identifying individuals affected by chronic stress; however, the reviews did not address whether these symptoms should be used as diagnostic criteria.

### 4.3 The multifaceted nature of clinical burnout

The narrow psychosocial conceptualization of burnout depicted in the included reviews stands in contrast to the broader literature on chronic stress, and raises important questions about whether clinical burnout, seen as a severe consequence of chronic stress, should be conceptualized solely as a work-related condition. This is particularly relevant given that, based on the conceptualizations found in the included reviews, chronic stress is understood to arise from both work-related and non-work-related sources.

Although leading researchers in the field of work-related stress and burnout recognize that stress originates from both work and non-work domains (Bianchi et al., 2014; Demerouti and Bakker, 2022; Bianchi and Schonfeld, 2024; De Witte and Schaufeli, 2025), prevailing burnout and job strain instruments remain anchored in work-centric theories like the Job Demands-Resources (JD-R) and Effort-Reward Imbalance models. While valuable for mapping workplace dynamics and employee well-being, these frameworks lack diagnostic utility for clinical burnout and fail to explain how work-related stress escalates into severe chronic symptoms, showing inconsistent predictive power, with job resources often inadequately mitigating demand impacts (Taris, 2006; Häusser et al., 2010; Guthier et al., 2020; Gonzalez-Mulé et al., 2021). This suggests such models overlook critical non-work stressors and biological mechanisms essential to understanding clinical burnout’s complexity (Van Dam, 2021; Khammissa et al., 2022).

### 4.4 System dynamics perspective

Existing clinical burnout assessments typically rely on isolated indicators, either psychosocial scales or single biomarkers. This fragmented strategy does not acknowledge the condition’s multidimensional nature and therefore prevents the development of a coordinated, clinically usable assessment. To move beyond a single-factor view, a system-dynamics perspective can clarify complex, dynamic phenomena. In the context of climate change, for instance, system dynamics modeling is increasingly used to assess tipping points, by modeling feedback loops between global warming, economic stability, policy interventions, and public behavior, revealing how emissions or policies can either catalyze financial instability or enable successful transitions (Lamperti et al., 2025). Just as system dynamics modeling helps uncover tipping points and transitions in climate systems, it can also clarify complex health dynamics. In stress research, where causal links are often represented by a single cause loop (CLD), a systems approach better captures the interplay of biological, psychological, and social factors over time, showing how their interaction drives health outcomes (Crielaard et al., 2024). The temporal delays and nonlinear trajectories observed in the development of clinical burnout underscore the relevance of system dynamics modeling, which is well-suited to capturing complex, time-evolving processes. As Lehman et al. (2017) argue, health emerges from dynamic, reciprocal interactions whose influence can vary within individuals over time. This is supported by empirical findings: Van Laethem et al. (2016) demonstrated how delayed effects, such as sleep loss exacerbating stress through rumination, contribute to cumulative strain, while Veldhuis et al. (2020) identified nonlinear symptom escalation and tipping points, suggesting that burnout can unfold in abrupt and disproportionate ways. Together, these findings highlight the value of a dynamic systems approach to understanding, diagnosing, and treating clinical burnout. These findings underscore the methodological value of system dynamics modeling, aligning closely with the network theory of mental disorders by Borsboom (2019). Rather than interpreting symptoms as the result of a single underlying cause, both perspectives conceptualize disorders as emergent outcomes of dynamic, reciprocal interactions and feedback loops among symptoms. Correspondingly, physiological biomarkers might be better understood not as fixed diagnostic indicators with universal cutoff values, but as dynamic variables that interact with psychological, social, and contextual factors involved in the onset and maintenance of biopsychosocial stress-related disorders. This conceptual integration is illustrated in Box 1, which applies system dynamics modeling to the case of clinical burnout.

**BOX 1 Application of system dynamics to clinical burnout.**

Applying system dynamics theory to clinical burnout might look like this: acute stressors (e.g., workload) trigger elevated cortisol and ANS activity, amplifying perceived stress, particularly in individuals with prior adversity, further elevating cortisol in a reinforcing loop. Sustained stressors (e.g., caregiving, ruminating, worrying thoughts) then drive progression to hypercortisolism (HPA-axis dysregulation), undermining resilience. This chronic dysregulation suppresses immunity (elevated CRP) and drains recovery capacity (lowered HRV), fueling escalating symptoms (fatigue, cognitive/sleep dysfunction) further dysregulating biological systems (HPA-axis exhaustion through hypocortisolism). Accumulated dysfunction than nears a *tipping point* (e.g., depression, autoimmune risk), where minor added stressors may trigger severe health collapse.

The system dynamics framework captures reciprocal feedback loops, showing how physiological dysregulation both results from and contributes to chronic stress, potentially creating new stressors and reinforcing the burnout cycle. Rather than serving as standalone diagnostic tools, biomarkers, and psychosocial (self-rated) scales can help explain how chronic stress translates into health deterioration over time within individuals, highlighting the importance of tracking changes within the person rather than applying population-level thresholds. Building on this need for a broader and multifactorial conceptualization, adopting a system dynamics perspective fundamentally could enhance our understanding of clinical burnout’s nature, especially in contrast to traditional static measurements.

### 4.5 Chronic stress syndrome (CSS) as an alternative label for clinical burnout

While the system dynamics approach offers conceptual value by framing clinical burnout as a health condition shaped by reciprocal feedback loops between environmental and internal stressors, biological responses, and psychological factors, the absence of a standardized clinical definition limits its diagnostic application within such a framework. Clarifying the fundamental nature of clinical burnout is therefore essential to advance causal understanding, refine diagnosis, and develop effective interventions.

Although organizational psychologists and clinicians widely acknowledge burnout, particularly its severe clinical manifestation (Van Dam, 2021; Schaufeli et al., 2023a; Demerouti and Bakker, 2025), the term itself may inadequately reflect the condition’s severity and clinical nature. Current conceptualizations seem to neglect research, including the findings of this scoping review, demonstrating chronic stress’s multifaceted biopsychosocial character, involving sustained biological dysregulation alongside psychological and social processes. Thus, the clinical condition termed “burnout” may be better reframed as Chronic Stress Syndrome (CSS): an autonomic disorder triggered by prolonged stress exposure in any life domain, marked by systemic neuroendocrine dysregulation and psychosocial impairment (e.g., sleep impairments, cognitive dysfunction, persistent exhaustion) (Van Zweden, 2015). CSS would reflect the complexity of sustained stress exposure, distinguishing it from disorders where stress is merely contributory (e.g., depression), and should be framed explicitly within a biopsychosocial model.

### 4.6 Strengths and limitations

This scoping review offers several methodological strengths. By situating burnout within the broader context of chronic stress, it captures a wide spectrum of literature and enables a structured mapping of how stress is conceptualized and measured. This approach not only clarifies the diverse constructs and operationalizations of chronic stress but also positions burnout within that framework, highlighting its theoretical underpinnings and measurement challenges. In doing so, the review provides a comprehensive knowledge base essential for the development of valid and multidimensional diagnostic criteria for clinical burnout. Furthermore, by exclusively including review articles, this study synthesizes scientific literature across a broad range of populations, methodologies, and measurement tools, enhancing the generalizability and relevance of the findings.

A potential limitation of this review is its exclusive focus on review articles, which may have led to the omission of individual primary studies that offer valuable insights. This is particularly relevant for studies that report promising findings on the diagnostic accuracy or discriminative value of specific assessment tools used to measure chronic stress or to differentiate between individuals with clinical burnout, subclinical symptoms, or healthy controls. As such, some recent or methodologically rigorous studies, especially those not yet synthesized in reviews, may have been excluded, potentially narrowing the scope of emerging evidence in this area.

A limitation of this review is the heterogeneity of the investigated burnout populations across the included studies. Most studies assessed burnout symptoms in otherwise healthy working individuals, while only a few examined clinical populations with severe symptoms and prolonged recovery. Because of this variation, the findings of this scoping review should be interpreted with caution when applied to clinical populations.

Another limitation of this scoping review is that no formal quality assessment of the included studies was conducted. This approach aligns with the PRISMA-ScR and JBI methodological frameworks, which do not require critical appraisal, as the primary aim of a scoping review is to map and summarize the existing research landscape rather than evaluate study quality. However, the absence of a quality assessment means that potential differences in methodological rigor across studies could not be accounted for, which should be considered when interpreting the findings.

## 5 Conclusion

Although chronic stress is widely recognized as a major contributor to clinical burnout, this scoping review demonstrates the academic field lacks a unified definition of chronic stress, nor does it provide robust and valid instruments to either measure chronic stress or diagnose clinical burnout. In term of conceptualization, definitions of chronic stress vary widely, ranging from prolonged exposure to external or internal stressors, to sustained physiological and psychological responses, to adverse health outcomes such as clinical burnout.

The biopsychosocial model emerges as the most frequently applied framework for understanding how chronic stress affects health, viewing stress as a multidimensional process shaped by the interplay between biological mechanisms (e.g., neuroendocrine responses), psychological factors (e.g., coping, emotion regulation), and social contexts (e.g., social economic status, work environment).

Across the reviewed studies, measurement approaches for assessing chronic stress, varied widely, reflecting the diversity in how chronic stress is conceptualized and defined. In evaluating their discriminative value, it appears to be limited, as no single biomarker or questionnaire demonstrates sufficient precision to serve as a standalone diagnostic tool for clinical burnout.

Despite the absence of a single diagnostic biomarker for clinical burnout, the findings of this review support the biopsychosocial model of chronic stress, demonstrating that findings across the included reviews consistently linked chronic stress to dysregulation across multiple physiological systems, including the HPA axis (hyper- and hypocortisolism), immune function (e.g., elevated CRP levels, suppression of global immunity), cardiovascular and ANS indicators (e.g., elevated heart rate and blood pressure, reduced HRV), the allostatic load index, and promising new biomarkers related to cellular aging (mitochondria and telomere length) and epigenetics (DNA methylation and miRNAs).

These integrated findings underscore the importance of viewing chronic stress not as a purely psychosocial phenomenon, but as a systemic condition with measurable physiological correlates. Despite being closely related to chronic stress, clinical burnout continues to be understood mainly within a psychosocial framework overlooking the biological dysregulation that may form part of its etiology. A more promising way to understand and diagnose clinical burnout may therefore lie in system dynamics modeling, which can capture the complex and interacting pathways between psychosocial, biological, and behavioral factors within a dynamic, non-linear, and time lagged system, rather than relay on any single measurement tool. This system dynamics perspective enables researchers and clinicians to examine how stress-related mechanisms and diagnostic thresholds evolve over time, across contexts, and within individuals. It also underscores the need to integrate biological aspects of the stress response into the diagnosis and treatment of chronic stress related disorders, moving beyond approaches that conceptualize stress exclusively in psychosocial terms.

## Data Availability

The original contributions presented in the study are included in the article/Supplementary material, further inquiries can be directed to the corresponding author.
