# Supplementary material for: Chronic stress in relation to clinical burnout: an integrative scoping review of definitions and measurement approaches
Source: Front Psychol. 2025 Dec 12;16:1712340. doi: 10.3389/fpsyg.2025.1712340 (PMC12740940; doi:10.3389/fpsyg.2025.1712340)
Supplement: Supplementary file 1 [file Data_Sheet_1.docx]

The literature search was conducted on April 9^th^, 2024, and updated on July 8^th^ 2025.

**Pubmed**

("Burnout, Professional"[Mesh] OR "Occupational Stress"[Mesh] OR "chronic stress"[tiab:~3] OR burn-out*[tiab] OR burnout*[tiab] OR exhaustion-disorder*[tiab] OR workrelated-stress*[tiab] OR work-related-stress*[tiab] OR occupational-stress*[tiab] OR work-stress*[tiab] OR workplace-stress*[tiab] OR job-stress*[tiab]) **AND** ("Biomarkers"[Mesh] OR "Allostasis"[Mesh] OR "Psychometrics"[Mesh] OR biomark*[tiab] OR biological-mark*[tiab] OR physiological*[tiab] OR psychological*[tiab] OR psychosocial*[tiab] OR biopsychosocial*[tiab] OR psycho-social*[tiab] OR biopsycho-social*[tiab] OR bio-psycho-social*[tiab] OR heart-rate-variabil*[tiab] OR biological[tiab] OR biology[ti] OR allostatic-load*[tiab] OR allostases[tiab] OR allostasis[tiab] OR psychometric*[tiab] OR psycho-metric*[tiab]) **AND** ("Surveys and Questionnaires"[mesh] OR "Biomarkers"[Mesh] OR measure*[tiab] OR assessment*[tiab] OR instrument[tiab] OR instruments[tiab] OR questionnaire*[tiab] OR survey*[tiab] OR identif*[tiab] OR tool[tiab] OR tools[tiab] OR indicator*[tiab] OR biomark*[tiab] OR biological-mark*[tiab]) **AND** ("Review" [Publication Type] OR "Systematic Review" [Publication Type] OR "Meta-Analysis" [Publication Type] OR "systematic review"[tiab:~3] OR "systematic reviews"[tiab:~3] OR meta-analys*[tiab] OR metaanalys*[tiab] OR review[ti]) **NOT** ("Oxidative Stress"[MJ] OR oxidative-stress*[tiab])

**Medline (EBSCOhost)**

(MH "Burnout, Professional+" OR MH "Occupational Stress+" OR TI(((chronic* OR occupational* OR work* OR job*) N3 (stress*)) OR burn-out* OR burnout* OR ((exhausti*) N3 (disorder*))) OR AB(((chronic* OR occupational* OR work* OR job*) N3 (stress*)) OR burn-out* OR burnout* OR ((exhausti*) N3 (disorder*)))) **AND** (MH "Biomarkers+" OR MH "Allostasis+" OR MH "Psychometrics+" OR TI(biomark* OR biological*-mark* OR physiological* OR psychological* OR psychosocial* OR biopsychosocial* OR psycho-social* OR biopsycho-social* OR bio-psycho-social* OR heart-rate-variabil* OR biological OR allostatic-load* OR allostases OR allostasis OR psychometric* OR psycho-metric* OR biology) OR AB(biomark* OR biological*-mark* OR physiological* OR psychological* OR psychosocial* OR biopsychosocial* OR psycho-social* OR biopsycho-social* OR bio-psycho-social* OR heart-rate-variabil* OR biological OR allostatic-load* OR allostases OR allostasis OR psychometric* OR psycho-metric*)) **AND** (MH "Surveys and Questionnaires+" OR MH "Biomarkers+" OR TI(measure* OR assessment* OR instrument OR instruments OR questionnaire* OR survey* OR identif* OR tool OR tools OR indicator* OR biomark* OR biological*-mark*) OR AB(measure* OR assessment* OR instrument OR instruments OR questionnaire* OR survey* OR identif* OR tool OR tools OR indicator* OR biomark* OR biological*-mark*)) **AND** (PT "Review" OR PT "Systematic Review" OR PT "Meta-Analysis" OR TI(((systematic*) N3 (review*)) OR meta-analys* OR metaanalys* OR review*) OR AB(((systematic*) N3 (review*)) OR meta-analys* OR metaanalys*)) **NOT** (MM "Oxidative Stress" OR TI(oxidative-stress*) OR AB(oxidative-stress*))

**Embase**

('chronic stress'/exp/mj OR 'burnout'/exp/mj OR 'job stress'/exp/mj OR (((chronic* OR occupational* OR work* OR job*) NEAR/3 (stress*)) OR burn-out* OR burnout* OR ((exhausti*) NEAR/3 (disorder*))):ab,ti,kw) **AND** ('marker'/exp/mj OR 'biological marker'/exp/mj OR 'psychometry'/exp/mj OR 'heart rate variability'/exp/mj OR 'allostatic load'/exp/mj OR (biomark* OR biological*-mark* OR physiological* OR psychological* OR psychosocial* OR biopsychosocial* OR psycho-social* OR biopsycho-social* OR bio-psycho-social* OR heart-rate-variabil* OR biological OR allostatic-load* OR allostases OR allostasis OR psychometric* OR psycho-metric*):ab,ti,kw OR (biology):ti) **AND** ('questionnaire'/exp OR 'biological marker'/exp OR (measure* OR assessment* OR instrument OR instruments OR questionnaire* OR survey* OR identif* OR tool OR tools OR indicator* OR biomark* OR biological*-mark*):ab,ti,kw) **AND** (review:it OR ((((systematic*) NEAR/3 (review*)) OR meta-analys* OR metaanalys*):ab,ti) OR (review):ti) **NOT** ('oxidative stress'/exp/mj OR (oxidative-stress*):ab,ti,kw)

**PsycINFO (EBSCOhost)**

(DE "chronic stress" OR DE "burnout" OR DE "occupational stress" OR TI(((chronic* OR occupational* OR work* OR job*) N3 (stress*)) OR burn-out* OR burnout* OR ((exhausti*) N3 (disorder*))) OR AB(((chronic* OR occupational* OR work* OR job*) N3 (stress*)) OR burn-out* OR burnout* OR ((exhausti*) N3 (disorder*)))) **AND**(DE "biological markers" OR DE "psychometrics" OR DE "classical test theory" OR DE "consistency (measurement)" OR DE "error of measurement" OR DE "external validity" OR DE "factor analysis" OR DE "internal validity" OR DE "item analysis (test)" OR DE "item response theory" OR DE "measurement invariance" OR DE "measurement models" OR DE "multivariate analysis" OR DE "test construction" OR DE "test reliability" OR DE "test sensitivity" OR DE "test specificity" OR DE "test validity" OR DE "variability measurement" OR TI(biomark*  OR biological*-mark* OR physiological* OR psychological* OR psychosocial* OR biopsychosocial* OR psycho-social* OR biopsycho-social* OR bio-psycho-social* OR heart-rate-variabil* OR biological OR allostatic-load* OR allostases OR allostasis OR psychometric* OR psycho-metric* OR biology) OR AB(biomark*  OR biological*-mark* OR physiological* OR psychological* OR psychosocial* OR biopsychosocial* OR psycho-social* OR biopsycho-social* OR bio-psycho-social* OR heart-rate-variabil* OR biological OR allostatic-load* OR allostases OR allostasis OR psychometric* OR psycho-metric*)) **AND**(DE "surveys" OR DE "consumer surveys" OR DE "mail surveys" OR DE "online surveys" OR DE "telephone surveys" OR DE "questionnaires" OR DE "biological markers" OR TI(measure* OR assessment* OR instrument OR instruments OR questionnaire* OR survey* OR identif* OR tool OR tools OR indicator* OR biomark* OR biological*-mark*) OR AB(measure* OR assessment* OR instrument OR instruments OR questionnaire* OR survey* OR identif* OR tool OR tools OR indicator* OR biomark* OR biological*-mark*)) **AND** ((MD 0800 OR MD 0830 OR MD 1200 OR TI(((systematic*) N3 (review*)) OR meta-analys* OR metaanalys* OR review*) OR AB(((systematic*) N3 (review*)) OR meta-analys* OR metaanalys*)) **NOT** (MM "oxidative stress" OR TI(oxidative-stress*) OR AB(oxidative-stress*))
