# Supplementary material for: Chronic stress in relation to clinical burnout: an integrative scoping review of definitions and measurement approaches
Source: Front Psychol. 2025 Dec 12;16:1712340. doi: 10.3389/fpsyg.2025.1712340 (PMC12740940; doi:10.3389/fpsyg.2025.1712340)
Supplement: Supplementary file 2 [file Data_Sheet_2.docx]

| **Author** | **Type of review** | **Included studies** | **Conceptualization of Chronic stress** | **Explanatory model** | **Population** | **Stress measurement tools** | **Key findings (predictive value)** |
| --- | --- | --- | --- | --- | --- | --- | --- |
| Aguayo-Estremera et al. (2024) | Meta-analysis | 45 | Burnout | Psychosocial model | Working population, teachers, medical professionals, other human service professionals | Psychosocial scales:  MBI | Due to variable reliability, errors may occur when assessing the accuracy of burnout diagnoses. |
| Allen et al. (2017) | Systematic Review | 151 | Caregiving | Biopsychosocial model | Informal dementia caregivers (spouses / children / siblings) | Biomarkers related to:   - HPA-axis activity - immune system - cardiovascular measures - neurocognition and neurotrophins   Psychosocial scales:  Caregiving yes/no  Behavioral scales:  Cognitive performance tests, Sleep polysomnography, actigraphy | Dementia caregiving is related to increased cortisol and poorer cognitive performance (attention and executive function), associations with immune system activation were mixed. |
| An et al. (2016) | Systematic Review | 40 | Caregiving, SES, Discrimination, Neighborhood violence, Psychiatric disorders (depression / anxiety, fear of dying), CVD, Acute myocardial infarction, Obesity, Social Isolation,  Work-related stress, Shift work, Burnout, Loneliness, Unspecified (chronic) stress, psychological ill-being, Aging, Noise | Biopsychosocial model | Healthy people in various age categories (18-65), white vs black, children with hypertension (HT) and their mothers, depressed people, central venous catheter (CVC) patients, paramedics, police officers, patients admitted with Acute coronary syndrome (ACS), volunteers, caregivers, male coronary artery disease patients (CAD), patients with acute myocardial infarction (AMI), woman free of cardiometabolic risk factors, woman employed in fulltime jobs, cardiovascular disease patients (CVD), people with chronic heart failure (CHF) | Biomarkers related to:   - HPA-axis activity - immune system   Psychosocial scales:  Not specified | Salivary cortisol is consistently associated with chronic psychosocial stress. |
| Bakusic et al. (2017) | Systematic Review | 19 human studies | Burnout, Chronic stress, Work related stress, Depression | Biopsychosocial model | Shift working nurses (f), Japanese manufacturing company workers (9%f), Healthy adults, Lifetime depression history, Japanese hospital patients, Twin- and adoption studies, People with a depression | Biomarkers related to:   - DNA methylation   Psychosocial scales:  Chronic stress: TICS  Burnout: MBI-GS  Work related stress: JCQ  Depression: BDI, SSAGA, MINI, SCID | Chronic stress, Burnout and work stress were significant contributors to higher methylation levels in the HPA axis related genes. |
| Chida et al. (2009) | Systematic Review and Meta-analysis | 147 | Job stress, General (non-work) life stress, Burnout / Exhaustion, Psychiatric disorders | Biopsychosocial model | Not specified | Biomarkers related to:   - Cortisol awakening response (CAR)   Psychosocial constructs were assessed using various validated tools, including (among others):  Job Stress: ERI, JDC, DC, Work engagement, UWES, Overcommitment, Time pressure, Overload, TICS  Burnout: MBI, SMBQ, Exhaustion/Fatigue: CIS, Chronic Fatigue Syndrome criteria, ESM/EMA (state measures), Fatigue Diary  General Life stress: PSS, SEI, Daily Hassles, PERI Life Events, CBCL, CTQ, CTES  Depression: BDI, CES-D, CDI, MDIS, EPDS, DISH, HDI  Anxiety: STAI, MASC, RCADS, Penn State Worry Questionnaire, Diary ratings (e.g., threat, fatigue)  PTSD: CAPS, SCID | Job stress and general life stress are linked to an increased cortisol awakening response (CARi), while fatigue, burnout, or exhaustion and posttraumatic stress syndrome are associated with a reduced CARi. |
| Coimbra et al. (2020) | Systematic Review | 12 | Social discrimination | Biopsychosocial model | African American, White, Mexican, Hispanic Mexican American | Biomarkers:   - Telomere length   Psychosocial scales:  Everyday Discrimination Scale (EDS), Implicit Association Test for racial bias, Major Experiences of Discrimination, Experiences of Discrimination (EOD), Gender and Racial Discrimination (GRD), Source of Discrimination (SOD), Lifetime Burden of Discrimination (LBD) | There is weak evidence for a direct association between social discrimination and telomere length, as studies included in the review showed mixed results. |
| Danhof-Pont et al. (2011) | Systematic Review | 31 | Clinical burnout | Biopsychosocial model, emphasizing both biological mechanisms and psychological symptoms of burnout | Healthy workers, Burnout patients recruited through occupational health practitioners or through registers of an insurance company, Burnout patients from a clinic or different institutions, Patients of a fertility clinic, Teachers, Managers, Healthy men | Biomarkers related to:   - HPA-axis activity - ANS function - Metabolic system - Immune system - Other hormones - Antioxidants   Psychological scales:  MBI, SMBM, psychological diagnosis DSM IV adjustment disorder, neurasthenia (ICD-10), somatoform disorder, adaptation disorder  Behavioral scales:  Sleep recordings | No convincing relationship was found between any of the 38 biomarkers investigated and burnout. |
| De Beer et al. (2024) | Systematic Review and Meta-Analysis | 35 | Burnout | Psychosocial model, workplace stress leads to burnout | Employees | Psychosocial scale:  MBI-GS | The MBI shows moderate criterion validity, especially concerning depersonalization (DE) and personal accomplishment (PA) . The paper advises against using the tool for diagnostic medical conditions, limiting its clinical utility. |
| De Looff et al. (2018) | Systematic Review | 38 | Job stress, Burnout | Biopsychosocial model | Singapore police officers, CVD susceptible nurses, Newly employed female nurses, Civil servants, Healthy male factory workers, Healthy employed day shift working men, Male managers of a Dutch telecom company, Employees of IT company, White collar workers in the public administration, Working population, Workers of a chemical company, Full time employed men, Health professionals and office clerks, Junior doctors, Resident doctors, White collar workers, Blue collar workers, Employed people working full time, Mannheim Industrial Cohort, Qualified nurses, Employed population, former ED patients, Male shipyard workers, Female nurses, First-aid doctors, Employees of consumer goods company, Employed working and on sick leave burnout patients, Employees from an airplane manufacturer, Soldiers, Full-time school teachers, Employees from IT company, Full-time employed men, Male personnel at a petrochemical plant, Employed hypotensive and hypertensive men, Healthy female nurses, Women working in Budapest, Employed people, Healthy hospital workers, Shift workers, daytime workers, White collar workers of a computer company | Biomarkers related to:   - ANS function (HRV) - Cardiovascular measures (heart rate, skin conductance (EDA))   Psychosocial scales:  Job stress: JDC, ERI, OI  Burnout: MBI, SMBQ, CBI | Higher levels of job stress are associated with lower HRV and elevated heart rate (HR).  The relation with burnout was less clear. |
| Deligkaris et al. (2014) | Systematic Review | 15 | Burnout | Psychosocial model, mainly based on the Conservation of Resources Theory (COR) | Burnout outpatients, most of whom were on sick leave, Individuals with high burnout scores who were still working, high vs low burnout scores healthy controls, non-clinical populations, health care workers, psychiatric nurses, military personnel, anesthetists | Psychosocial scales:  MBI-GS, SMBM, Burnout Tedium measure  Cognitive function: objective psychometric tests | This review shows that there is an association between burnout and cognitive impairment (executive functions, attention and memory). |
| Dowd et al. (2009) | Literature Review | 26 | SES | Biopsychosocial model | Nationally representative (non-institutionalized) populations | Biomarkers related to:   - HPA-axis activity (Cortisol, CAR, Diurnal cortisol slopes) - Allostatic Load index (different neuroendocrine, metabolic, immune and cardiovascular markers)   Psychosocial scales:  Employment, Parental education, income,  duration of poverty,  wealth, family Income  occupational grade, subjective social status, financial strain, Material hardship, neighborhood, assets, father, adult, lifetime social class | The relationship between SES and cortisol were mixed, with little evidence that lower SES is related to higher levels of cortisol. Lower SES was more strongly associated with higher levels of allostatic load (AL), particularly through cardiovascular and metabolic markers, rather than neuroendocrine components. |
| Emal et al. (2023) | Systematic Review | 17 | Psychological Distress in healthcare workers | Psychosocial model, psychological distress as a response to work demands, identifying effort-reward and job demands as contributors. | Health care professionals (e.g., Nurses, Physicians, Psychologists, Social workers) | Psychosocial scales: Work-functioning screener-healthcare (WFS-H), Burnout Battery, Physician Wellbeing Index (PWBI), Professional Quality of Life (ProQOL), Burnout-thriving index, Single-item burnout,  Professional fulfilment index (PFI) | Given their low methodological quality and the paucity of validation studies, these instruments cannot reliably screen or diagnose psychological distress in healthcare workers and are therefore not recommended for clinical use in burnout assessment. |
| Figueiredo et al. (2024) | Systematic Review | 5 | Burnout | Psychosocial model | Health care workers | Psychosocial scales:  SMBM, SMBQ | The SMBM should not be utilized for clinical purposes due to insufficient content validity. |
| Finlay et al. (2022) | Systematic Review | 25 | Adverse Childhood Experiences (ACE’s) | Biopsychosocial model (with a primary emphasis on allostatic load and physiological dysregulation) | Nationally representative populations. | Biomarkers related to:   - Allostatic Load (AL) index (6-24 different neuroendocrine, metabolic, immune and cardiovascular markers)   Psychosocial scales:  Childhood trauma  Household dysfunction  Infant separation  Childhood maltreatment  Low SES  Social Isolation  Childhood SES, poverty  Childhood abuse (emotional and physical)  Neglect  Experience of childhood (on a scale)  Early life trauma  Childhood discrimination  Childhood sexual abuse | Across multiple studies, adolescents and adults with more ACEs consistently show higher AL indices compared to healthy controls.  Allostatic load is a useful tool for assessing current health, early detection of health problems, and identifying those at risk. |
| Grossi et al. (2015) | Review | 59 | Clinical burnout / Exhaustion disorder | Biopsychosocial model, integrating biological and psychological aspects of burnout to explain stress-health relationships | Burnout patients, patients with Exhaustion Disorder | Biomarkers related to:   - HPA-axis activity - immune system - cardiovascular measures - the metabolic system - Prefrontal volume - Hippocampal volume   Psychological scales:  MBI, CBI, BM, OLBI, SMBM, SMBQ, KES, KEDs, s-ED, Burnout Measure (BM), psychological diagnosis DSM IV adjustment disorder, anxiety, mood (axis I), ICD-10 Exhaustion Disorder, ICD Neurasthenia  Behavioral scales: sleep disturbances (Polysomnographic recordings at home  Electronic diaries), cognitive function (Measures of general cognitive ability and verbal memory. Tests of working memory and reaction time.  Subjective tests of cognitive impairments. fMRI) | No biomarker for clinical burnout or exhaustion disorder (ED) was found.  Sleep impairments, especially insomnia-type disturbances, were found to be important causative and maintaining factors for clinical burnout and ED.  Cognitive impairments in memory and executive function were associated with ED. |
| Guidi et al. (2021) | Systematic Review | 267 | Sociodemographic factors (SES, discrimination), Work-related stress, Burnout, Early life events, Caregiving, Aging, Well-being and coping strategies, Lifestyle habits, CVD, Diabetes, Gynecology and obstetrics, musculoskeletal disorders, cancer, periodontal diseases, mood and anxiety disorders, PTSD, Psychotic disorders, Alcohol dependence | Biopsychosocial model, integrating biological mechanisms with psychological and social factors to explain stress-health relationships | Not specified | Biomarkers related to:   - Allostatic Load (AL) index (exact biomarkers were not mentioned in this paper) | Allostatic load tends to increase in individuals with lower SES, poor neighborhood conditions, limited education, and experiences of racial discrimination. Studies also link high AL to work-related stress (including caregiving) and a higher risk of burnout. Moreover, adverse childhood experiences, such as abuse or maltreatment, predict elevated AL in adulthood. |
| Hagan et al. (2024) | Systematic Review | 17 | Burnout | Psychosocial model | Physicians, Trainees, Anesthesia personnel, Primary care staff, medical students, Oncologists, General practice physicians, Surgeons, Internal Medicine residents, Alumni of School of Medicine. | Psychosocial scales:  Singe Item Burnout Question (SIBQ) | The SIBOQ can identify subgroups with high or low burnout, but it lacks sufficient reliability for comparing results between context or for precise diagnostic purposes. |
| Järvelin-Pasanen et al. (2018) | Systematic Review | 10 | Job stress | Biopsychosocial model, using both psychosocial models like the Effort-Reward Imbalance and the Job-Demand Control theory and biological mechanisms like ANS activation to explain stress-health relationships | Healthy employed individuals, nurses, hospital workers, physicians, factory workers, media workers, workers in consumer goods production, worker in airplane manufacturing, white-collar workers. | Biomarkers:   - ANS function (HRV)   Psychosocial scales:  Job stress: JCQ, ERI, JSQ, OSQ | Heightened occupational stress was found to be associated with lowered HRV. |
| Johnson et al. (2013) | Systematic Review | 40 | Work-related stress, Burnout, Unemployment, Caregiver stress, Interpersonal stress, SES, Discrimination | Biopsychosocial model | Not specified | Biomarkers:   - Immune system: C- Reactive Protein (CRP)   Psychosocial scales:  Work-related stress: ERI, JCQ, subjective job strain, salutogenic subjective work analysis.  Burnout / vital exhaustion: MBI, SMBM, SMBQ, Shortened Maastricht Exhaustion questionnaire, Diagnostic criteria for Exhaustion Disorder  Unemployment: yes/no.  Caregiver: yes/no  Interpersonal stress: 12-item questionnaire, UCLA, Life Stress Interview, Personal interview  SES: Civil service employment grade, Registrar General’s Social Class, Father’s education, own education, own occupation, Income, education, Occupational status (manual lower, higher non-manual), Poverty and education, Individual SEP, community SEP, National Statistics, Socioeconomic classification, Education, occupational class, wealth, Education, employment, income, immigrant status, neighborhood characteristics (disorder and social capital), Crime (neighborhood burglary rate), income, education, Neighborhood characteristics (deprivation, problems, safety, cohesion)  Discrimination: Question: “Have you ever been discriminated against due to your race/ethnicity?”, Everyday Discrimination Scale, Experiences of Discrimination Index | Chronic psychosocial stress was found to significantly impact CRP elevations. |
| Johnson et al. (2017) | Systematic Review | 26 | SES | Biopsychosocial model, with a focus on allostatic load as a key mechanism linking stress to health outcomes | Healthy individuals of different nationalities | Biomarkers:   - Allostatic Load (AL) index, 59 biomarkers (ranged between 6 and 25, with a mode of 9)   Psychosocial scales: Employment, Parental education, income, Duration of poverty,  Education and wealth, Family Income, Occupational grade,  Subjective social status, financial strain, Material hardship, Neighborhood and family income, assets, Father, adult, lifetime social class | In almost all studies lower SES groups were found to have higher AL indices, with most studies not incorporating HPA-axis biomarkers of the neuroendocrine system. |
| Jonsdottir and Sjors Dahlman (2019) | Narrative Review | Not mentioned | Clinical burnout / Exhaustion Disorder | Biopsychosocial model, summarizing main endocrine and immune findings in relation to burnout | Burnout patients (clinical and non-clinical) | Biomarkers related to:   - HPA-axis activity - Immune system - Other hormones   Psychosocial scales:  MBI, SMBQ, psychological diagnosis DSM IV Neurasthenia, Exhaustion Disorder | No consistent evidence was found for reliable endocrinological, or immunological changes associated with burnout. |
| Kalliokoski et al. (2019) | Systematic Review | 20 human studies | Unemployment, Military training, SES, shift work, Caregiving, Chronic pain, post-birth complicates in neonates | Biopsychosocial stress | Not specified | Biomarkers:   - Hair Cortisol Concentrations (HCC)   Psychosocial scales:  No specified | Hair cortisol is an effective biomarker for recent or ongoing stress, but not for past stress (stressors that had passed during the time of sampling). |
| Kaltenegger et al. (2021) | Systematic Review and Meta-Analysis | 23 | Work-related stress | Biopsychosocial model, emphasizing biological mechanisms and workplace conditions | Adult employees within different occupational settings. | Biomarkers related to:   - Immune system (e.g., CRP, Fibrinogen, IFN-y, IL-1b, and TNF-a, IL-6, Leukocyte count)   Psychosocial scales:  JDC-S, OC, Job stress, social support | Results showed predominantly null and/or weak associations between working conditions and inflammatory biomarkers. However, workplace physical activity interventions significantly reduced inflammation, showing lowered CRP levels. |
| Kim et al. (2018) | Literature Review and Meta-Analysis | 37 | Psychological stressors (Perceived (chronic) stress, work-related stress, examination, Cumulative stress) | Biopsychosocial model | Not specified | Biomarkers:   - Heart rate variability (HRV)   Psychosocial scales:  Perceived job stressors  Scenario simulating a medical emergency, Stroop Word Color Conflict Test (STROOP), Workday versus non-workday, Self-rating of trait anxiety and perceived emotional stress, University examination, Computer work related mental stressor (in a laboratory setting), JCQ, ERI, Perceived mental stress during a workday, Job-strain questionnaire, (JSQ, State-trait anxiety inventory, Perceived stress scale, Cumulative stress / adversity checklist, Mental task, Self-reported chronic stress aspects (events, emotions, problems), Telephone interview, The medical students stress questionnaire | Most studies found that stress is associated with lowered HRV, primarily showing **reduced parasympathetic activity.** |
| Lindsäter et al. (2022) | Scoping Review | 89 | Exhaustion disorder | Biopsychosocial model, discussing both biopsychosocial and psychosocial models of stress | Individuals diagnosed with Exhaustion Disorder, burnout, or adjustment disorder | Biomarkers related to:   - HPA-axis activity - Growth factors - Cardiovascular measurements (e.g. HRV) - Allostatic load index (composite of 13 biomarkers) - Brain volumetric measures (e.g., MRI, fMRI, fNIRS)   Psychosocial scales:  Diagnostic criteria Exhaustion Disorder ICD-10 2005 diagnostic code F43.8A, KEDS, SMBQ-22, General Well-being index (PGWI), WHODAS 2.0.  Behavioral scales: cognitive function and sleep patterns | The KEDS has sensitivity and specificity above 95%, the SMBQ over 83% in separating clinical from healthy populations. Further validation is needed to their reliability in differentiating ED from other conditions.  No firm conclusions can be drawn regarding biological correlates that may be specific to exhaustion disorder. |
| Mathur et al. (2016) | Systematic Review and Meta-Analysis | 23 | Perceived stress | Biopsychosocial model | Adults, Combat veterans, Overweight or  obese females, Female breast cancer survivors, Caregivers of disabled children, Adults (some of whose mothers were psychologically stressed during pregnancy), Premenopausal mother (some with chronically ill child), Health females, Masto cytosis patients, fibromyalgia patients, coronary heart disease patients (many with major depression), females (formerly abused and non-abused), adults, females with sisters with breast cancer, post-menopausal woman (some dementia caregivers), patients with chronic pain from knee osteoarthritis | Biomarkers:   - Telomere length   Psychosocial scales:  Perceived stress scale (PSS), Calgary Symptoms of Stress Inventory (SOSI), Perceived Stress Questionnaire, Single-item measure | Higer Perceived stress over the past month was associated with reduced Telomere length. |
| McGee et al. (2023) | Systematic Review | 38 | Teacher stress | Biopsychosocial model, with a strong emphasis on biological mechanisms linking chronic stress to health outcomes | Teachers | Biomarkers related to:   - HPA-axis activity - Cardiovascular measures Cortisol - The immune system   Psychological scales:  Job stress: ERI, OC, Work-Demands Resources, JCQ, Job Strain, VBBA.  Burnout: Spanish Burnout Inventory, MBI, Psychological Distress Symptoms Checklist 90-R, Work engagement.  Perceived stress: TICS, PSS, Anxiety, Quality of Life, Daily Hassles, NFR | Chronic stress (burnout) is linked to blunted HPA responses and increased pro-inflammatory activity and decreased anti-inflammatory activity in the immune system. Acute stress (job strain) was positively related to increased HPA response. |
| Michel et al. (2022) | Meta-Analysis | 89 | Burnout | Psychosocial model, using the frameworks of COR and the JD-R model. Resource loss and job demands are components of stress which is related to burnout. | Working population (non-clinical) | Psychosocial scales:  SMBM | The validity of the SMBM as a diagnostic instrument in clinical practice remains unestablished. |
| Milaniak and Jaffee (2019) | Systematic Review and Meta-Analysis | 35 | Childhood SES | Biopsychosocial model, emphasizing inflammatory markers in relation to stress to explain stress-health relationships | General population (non-clinical), with individuals measured during childhood and during adulthood | Biomarkers related to:   - The immune system (CRP, IL-6, Fibrinogen)   Psychosocial scales:  Childhood socioeconomic status (SES) was measured using various indicators, including (but not limited to) parental education (such as maternal education or the highest level attained across both parents), parental occupation (for example, the father’s occupation or the head of household’s job during early years), and household income (including annual income or retrospective income reports at various child ages). Other measures included wealth indicators like homeownership, vehicle ownership, or the number of bedrooms per child in the household. | Low childhood socioeconomic status was associated with higher levels of inflammatory markers. However, the effect became non-significant when controlling for adult SES in longitudinal studies. |
| Murkey et al. (2022) | Systematic Review | 7 | SES, Racial discrimination and perceived chronic stress in African Americans | Biopsychosocial model with a strong emphasis on the weathering hypothesis, linking social and economic inequities to stress and health outcomes. | African American, Adults | Biomarkers:   - Telomere length - Allostatic Load index   Psychosocial scales:  Discrimination: African American yes/no  SES: Educational level, Economic hardship, Family socioeconomic risk, social mobility.  Perceived chronic stress:  Cumulative stress (childhood/adolescent), Life stress | Higher perceived chronic stress levels and experiences of racial discrimination are associated with shorter telomere length. Lower income and higher perceived chronic stress levels are associated with increased allostatic load among African Americans. |
| Noushad et al. (2021) | Systematic Review | 37 | Neonatal / postpartum stress in infants and mothers, SES, pregnant woman, maternal stress (caring for a child with obesity and having a disability), working conditions, burnout, physical and mental health disorders, woman who have gone through divorce, diabetes, caregivers, workers of pre-hospital emergency service, schoolteachers, premature infants, non-Hispanic black woman | Biological model with elements of the biopsychosocial model | Pregnant females, Infants, Healthy Adults, Burnout patients (clinical and non-clinical), Divorced woman, Diabetes patients, Hispanic and non-Hispanic women, Caregivers, Prehospitalary emergency service healthy workers, School teachers, Emergency physicians | Biomarkers related to:   - HPA-axis activity - The immune system - The metabolic system - Cardiovascular measures   Psychosocial scales:  Not specified | Cortisol, ACTH, BDNF, catecholamines, glucose, HbA1c, triglycerides, cholesterol, prolactin, oxytocin, DHEA-s, CRP and interleukins -6 and -8 were found to be potential biomarkers for chronic stress. |
| Oliveira et al. (2016) | Systematic Review | 18 | Caregiving, SES, Neighborhood adversity, ACE’s, Life stress | Biopsychosocial model | Caregiving for chronically ill children, Caregivers of Alzheimer’s disease patients, Caregivers of parent, a child or other friend/relative, Healthy adults, Convenience samples | Biomarkers:   - Telomere length   Psychosocial scales:  Caregiving: yes/no,  Neighborhood adversity: economic deprivation and perceived neighborhood disorder, Income to poverty ratio, social disadvantage (income/needs ratio), Poverty (income ratio),  ACE’s: Childhood maltreatment,  Adverse childhood difficulties, Number of childhood adversities including physical abuse, parental alcohol use, divorce, and separation from parents, Childhood trauma, such as physical neglect, family violence, physical abuse, forced sexual touching, or forced sexual intercourse, Family interpersonal violence and disruption, Separation from parents, physical/emotional traumatic experiences, Life Stress: Life course stress, Psychological aggression, sexual coercion, physical assaults, and injury experience, Domestic violence, frequent bullying victimization or physical maltreatment by an adult,  Trauma experiences during childhood and exposure to combat trauma during deployment | Evidence supports that chronic social stress accompanies telomere shortening, although methodological limitations prevent strong conclusions. |
| Ottaviani et al. (2016) | Systematic Review and Meta-Analysis | 60 | Perseverative cognition (rumination about the past and worry about the future) | Biopsychosocial model, with emphasis on the Perseverative Cognition Hypothesis | Healthy individuals | Biomarkers related to:   - HPA axis activity (cortisol) - ANS function (Blood pressure, Heart rate variability (HRV), Heart rate)   Psychosocial scales: state and trait measurements of perseverative cognition (worry and rumination) | Perseverative cognition was associated with increased heart rate, higher blood pressure, increased levels of cortisol, and lower heart rate variability. |
| Pate et al. (2023) | Review | 11 | Burnout | Psychosocial model, emphasizing appraisal and personal/environmental factors. Chronic stress is linked to burnout when stressors are unmitigated | Not specified | Psychosocial scales:  MBI, OLBI, CBI, BAT, Pro QOL, Single question burnout measurement, Perceived Stress Scale, Stress in context questionnaire, Stress overload scale | 11 Validated reliable surveys were identified for burnout research. The paper does not discuss diagnostic accuracy of burnout measurement scales. |
| Phillips et al. (2021) | Systematic Review | 18 | Early childhood stress, Racial ethnic stress, Workplace stress, Life stress | Biopsychosocial model, with an emphasis on HPA-axis activity as a mediator between chronic stress and health. | Mothers, Infants, Preschool children, At-risk children, African American adolescents, Indigenous young adults, University students, medical students, right-handed volunteers, Adults (employed), Middle aged workers, Adult cancer survivors, Acute Coronary syndrome adults, healthy controls, Bipolar disorder I patients, Adults with major Depressive disorder. | Biomarkers:   - Nail cortisol   Psychosocial scales:  At-risk children, pre-school children, African Americans from low-income background, Australian indigenous youth, Perceived Stress Scale (PSS), Strength and Difficulties Questionnaire | Nail cortisol may serve as a retrospective biomarker of chronic stress, with higher concentrations being associated with depression and acute coronary syndrome. |
| Picard and McEwen (2018) | Systematic Review | 6 | Adverse Childhood Experiences, Psychiatric disorders, Caregiving, Suicide attempts | Biopsychosocial model, highlighting mitochondria as both targets and mediators of stress. | American individuals experiencing adverse childhood experiences and with psychopathology (depression, anxiety), and substance abuse disorders compared with controls without ACE. Chines women with depression compared with controls without depression. | Biomarkers:   - Mitochondria   Psychosocial scales: ACE’s (cumulative score), Stressful Life Events (SLE), Suicide attempt through self-report Psychiatric disorders (PD) (SCID), Caregiving status (yes/no), Daily mood (pos/neg) | Chronic stressors influence mitochondrial functioning, causing significant changes in energy production capacity and morphology.  Even so, biomarkers alone are insufficient to assess chronic stress without functional measures. |
| Schaafsma et al. (2021) | Scoping Review | 22 | Work related stress, Burnout, Unemployment, Mental health problems | Biopsychosocial model, emphasizing both biological and psychological processes in linking chronic stress to health outcomes. | Working population, Employed versus Unemployed, Caregivers, Individuals with mental health, substance use, addiction and violence problems. | Biomarkers:   - Hair cortisol concentrations (HCC)   Psychosocial scales:  Work stressors: ERI, JCQ, Copenhagen Psychosocial Questionnaire, Survey about personal boundary violations at the workplace, shift work (yes/no), Emotional Labor Scale, Stressful Life Events Scale, Transformational Leadership Inventory, NFR  **Unemployment**: (yes/no),  **Burnout**: MBI  **Perceived Stress**: The Calgary Symptoms of Stress Inventory, TICS, PSS | The evidence for using hair cortisol (HCC) as a biomarker for chronic stress in occupational settings is inconsistent. |
| Segerstrom and Miller (2004) | Meta-Analysis | 300+ | Caregiving, Living with a handicap, Unemployment | Biopsychosocial model, emphasizing inflammatory markers and immune system indicators as mediators between stress and health outcomes. | Not specified | Biomarkers related to:   - The immune system   Psychosocial scales:  Not mentioned in this paper | Chronic stress triggers suppression of global immunity, which is a maladaptive response affecting long term health outcomes. |
| Shoman et al. (2021) | Systematic Review | 19 | Burnout | Psychosocial model, | Working population, General population, Psychologists, Population working in human service sector | Psychosocial scales:  MBI, Pines Burnout Measure (BM), PBM, OLBI, CBI | Among the five reviewed PROMs, the psychometric validity of the CBI was found to be the most valid, the OLBI followed, the MBI despite being widely used was found to have very low quality of evidence for its psychometric properties.  Even so there is no diagnostic standard for burnout, excluding the use of PROMs for Occupation Burnout as diagnostic tools in medical practice. |
| Shoman et al. (2022) | Systematic Review | 11 | Burnout | Psychosocial model, workplace stress that is not managed leads to burnout. The COR framework conceptualizing stress as the depletion of physical, emotional and cognitive energies. | Working population | Psychosocial scales:  SMBM, BAT | In terms of psychometric validity, the BAT was found to be superior to the SMBM. However, the quality of evidence for the BAT is still very low. The paper does not mention diagnostic accuracy. |
| Villacura-Herrera et al. (2025) | Meta-Analysis | 56 | Burnout | Psychosocial model | Employees, Healthcare workers, Teachers, Professionals, Administrative staff, Midwives, Recreational diving instructors, Middle school students, Police employees, Mental health employees, Emergency nurses, Civil servants, Physicians, Remote workers, Workers, Library employees, Nurses, General Practitioners, Bachelor students, Pharmacy professionals | Psychosocial scales:  BAT | The BAT is highly reliable for assessing burnout across different populations and settings. Even so the results warrant cautious interpretation in contexts requiring higher precision, such as diagnosing clinical burnout. |
| Walsh et al. (2021) | Systematic Review | 19 human studies | Caregiving, Social Isolation, Economic strain / low social status, Bereavement | Biopsychosocial model | Not specified | Biomarkers related to:   - The immune system   Psychosocial scales:  Not mentioned in this paper | Chronic stress is associated with an up regulation of pro-inflammatory gene transcription among peripheral monocytes, contributing to systemic inflammation |
| Wheeler et al. (2011) | Meta-Analysis | 84 | Burnout | Psychosocial model, implicitly associating chronic stress with the concept of burnout, linking severe work-related stress to health | Working population, medical professionals, Teachers | Psychosocial scales:  MBI | The MBI does not meet reliability standards for diagnostic use. The MBI can only classify burnout levels for research purposes. |
| Wiegand et al. (2017) | Review | 5 | Academic Exams | Biopsychosocial model, emphasizing the role of psychological stress in triggering responses related to the SAM and HPA axis. | Fourth grade medical students | Biomarkers:   - MicroRNAs   Psychosocial scales:  Spielberg’s State Trait Anxiety Inventory (STAI), Perceived Stress Questionnaire | A few miRNAs have shown altered expression in response to chronic stress. |
| Wosu et al. (2013) | Systematic Review | 39 | SES, Age, Sex, Race and ethnicity, Psychiatric disorders (Depression, Anxiety, PTSD, Bipolar), CVD, Cardio Metabolic Syndrome, Chronic Pain, Cushing’s syndrome, Adiposity, Pregnancy, Early Life Adversity, Lifestyle and behavioral factors, Cigarette smoking,  Oral contraceptive and medication intake, Physical activity | Biopsychosocial model, emphasizing both HPA axis activity and psychosocial aspects in linking stress to health outcomes | Clinical and non-clinical populations | Biomarkers:   - Hair Cortisol Concentrations (HCC)   Psychosocial scales:  Not mentioned in this paper | Hair cortisol (HCC) was found to be associated with stress-related psychiatric symptoms, Cushing’s syndrome, Shift work, physical activity, adiposity, substance abuse and pregnancy. |

Note: ACE = Adverse Childhood Experiences, ACTH = Adrenocorticotropic Hormone, ANS = Autonomic Nervous System, BAT = Burnout Assessment Tool, BDI = Beck Depression Inventory, BDNF = Brain-Derived Neurotrophic Factor, BM = Pines Burnout Measure, CAR = Cortisol Awakening Response, CAPS = Clinician-Administered PTSD Scale, CBCL = Child Behavior Checklist, CBI = Copenhagen Burnout Inventory, CDI = Child Depression Inventory, CES-D = Center for Epidemiologic Studies Depression Scale, CIS = Checklist Individual Strength, COR = Conservation of Resources, CRP = C-Reactive Protein, CTES = Childhood Traumatic Events Survey, CTQ = Childhood Trauma Questionnaire, CVD = Cardiovascular Disease, DC = Demand-Control Questionnaire, DHEA-s = Dehydroepiandrosterone Sulfate, DISH = Depression Interview and Structured Hamilton, ED = Exhaustion Disorder, EDA = Electrodermal Activity, EMA = Ecological Momentary Assessment, EPDS = Edinburgh Postnatal Depression Scale, ERI = Effort-Reward Imbalance Questionnaire, ESM = Experience Sampling Method, fMRI = Functional Magnetic Resonance Imaging, fNIRS = Functional Near-Infrared Spectroscopy, HbA1c = Glycated Hemoglobin, HDI = Hamilton Depression Inventory, HPA = Hypothalamic-Pituitary-Adrenal Axis, IFN-y = Interferon-Gamma Cytokine, IL-1b = Interleukin-1 Beta, IL-6 = Interleukin-6, JDC = Job Demand-Control Questionnaire, JDC-S = Job Demand-Control-Support Questionnaire, JCQ = Job Content Questionnaire, JD-R = Job Demand-Resources, JSQ = Job Stress Questionnaire, KED-s = Karolinska Exhaustion Disorder Scale, KES = Karolinska Exhaustion Scale, MASC = Multidimensional Anxiety Scale, MBI(-GS) = Maslach Burnout Inventory (General Survey), MDIS = Major Depression Inventory Scale, MINI = Mini International Neuropsychiatric Interview, MRI = Magnetic Resonance Imaging, NFR = Need for Recovery, OC = Organizational Commitment, OI = Organizational Injustice Scale, OLBI = Oldenburg Burnout Inventory, OSQ = Occupational Support Questionnaire, PBM = Professional Burnout Measure, PERI = Psychiatric Epidemiology Research Interview, PSS = Perceived Stress Scale, PTSD = Post-Traumatic Stress Disorder, RCADS = Revised Child Anxiety and Depression Scale, SAGA = Self-Assessment Goal Achievement, SAM = Sympathomedullary Pathway, SCID = Structured Clinical Interview for DSM-IV, s-ED = Self-Reported Exhaustion Disorder, SEI = Stress and Energy Inventory, SMBM = Shirom Melamed Burnout Measure, SMBQ = Shirom Melamed Burnout Questionnaire, STAI = State-Trait Anxiety Inventory, TICS = Trier Inventory for the Assessment of Chronic Stress, TNF-a = Tumor Necrosis Factor Alpha, UWES = Utrecht Work Engagement Scale, VBBA = Vragenlijst Beleving en Beoordeling van de Arbeid, WHODAS 2.0 = World Health Organization Disability Assessment Schedule 2.0.
